# PI3Kδ activity controls plasticity and discriminates between EMT and stemness based on distinct TGFβ signaling

**DOI:** 10.1038/s42003-022-03637-w

**Published:** 2022-07-25

**Authors:** Jean Agnetti, Vanessa Bou Malham, Christophe Desterke, Nassima Benzoubir, Juan Peng, Sophie Jacques, Souad Rahmouni, Emanuel Di Valentin, Tuan Zea Tan, Didier Samuel, Jean Paul Thiery, Ama Gassama-Diagne

**Affiliations:** 1 INSERM, Unité 1193, Villejuif, F-94800 France; 2 Université Paris-Saclay, UMR-S 1193, Villejuif, F-94800 France; 3 Université Paris-Saclay, UFR Médecine- INSERM UA9, Villejuif, France; 4 Laboratory of animal Genomics, GIGA-Medical Genomics, GIGA-institute, Université de Liège, Liège, Belgium; 5 Plateforme des vecteurs viraux, GIGA B34, GIGA-institute, Université de Liège, Liège, Belgium; 6 Cancer Science Institute of Singapore National University of Singapore, Center for Translational Medicine, 14 Medical Drive, #12-01, 117599 Singapore, Singapore; 7 AP-HP Hôpital Paul Brousse, Centre Hepato-Biliaire, F-94800 Villejuif, France; 8 Guangzhou Laboratory, International biological Island Guangzhou, 510005 Guangzhou, China

**Keywords:** Apicobasal polarity, Phosphoinositol signalling

## Abstract

The stem cells involved in formation of the complex human body are epithelial cells that undergo apicobasal polarization and form a hollow lumen. Epithelial plasticity manifests as epithelial to mesenchymal transition (EMT), a process by which epithelial cells switch their polarity and epithelial features to adopt a mesenchymal phenotype. The connection between the EMT program and acquisition of stemness is now supported by a substantial number of reports, although what discriminates these two processes remains largely elusive. In this study, based on 3D organoid culture of hepatocellular carcinoma (HCC)-derived cell lines and AAV8-based protein overexpression in the mouse liver, we show that activity modulation of isoform δ of phosphoinositide 3-kinase (PI3Kδ) controls differentiation and discriminates between stemness and EMT by regulating the transforming growth factor β (TGFβ) signaling. This study provides an important tool to control epithelial cell fate and represents a step forward in understanding the development of aggressive carcinoma.

## Abstract (web summary)

Overexpression of isoform δ of phosphoinositide 3-kinase (PI3Kδ) promotes stemness in hepatocellular carcinoma cell lines and mouse liver, whereas its inhibition promotes EMT, suggesting a key role for PI3Kδ in epithelial plasticity.

## Introduction

The class I phosphoinositide 3-kinases (PI3Ks) are the best-studied enzyme of PI-metabolism and frequently deregulated in cancer^1^. The class I PI3Ks consist of four isoforms (α β γ δ), however most of the fundamental or clinical studies were performed using pan-PI3K inhibitors targeting all isoforms. The isoform-specific roles just started to be investigated this last decade due to the generation of gene-targeted mice and commercially available isoform-selective inhibitors^2,3^. Isoform δ of phosphoinositide 3-kinase (PI3Kδ) is the latest member of class I PI3Ks identified more than 20 years ago as predominantly expressed in the spleen and thymus while almost undetectable in other tissues^4^.

PI3Kδ plays a major role in the immune system, and extensive work devoted to understanding this protein led to establishment of the first PI3K inhibitor, idelalisib (CAL-101), an ATP-competitive kinase inhibitor that targets PI3Kδ with a high potency and selectivity, approved for the treatment of lymphoma^5,6^. Nevertheless, the fundamental role of this enzyme in non-hematopoietic cells, notably those in the epithelium, remains enigmatic. Several recent studies have shown the level of this protein to be elevated in solid cancers^7–11^. Previously, we found that PI3Kδ plays an important role in epithelial cells using 3D cultured Madin Darby canine kidney (MDCK) cells^12^. In particular, we demonstrated that PI3Kδ is essential for correct polarization and hollow lumen formation. Indeed, pharmacological inhibition or knockdown of this enzyme led to an inverted polarity phenotype and a defect in extracellular matrix (ECM) assembly^12^. In the present study, we sought to determine the role of PI3Kδ in morphogenesis and plasticity in the context of the liver.

The most studied form of cellular plasticity is the epithelial to mesenchymal transition (EMT), a process through which epithelial cells lose their polarity and differentiation traits to acquire mesenchymal, then invasive characteristics^13–17^. EMT and its reverse process mesenchymal-epithelial transition (MET) represent key mechanisms in embryonic development and are essential driver of plasticity in cancer and resistance to treatments^14,18,19^. Number of signaling pathway including transforming growth factor-β (TGFβ), Wnt, Notch, Hedgehog and Hippo pathways contributed to EMT. Furthermore, remodeling of the extracellular matrix (ECM) and changes to cell interactions with the ECM are essential regulators of EMT^20^. The connection between the EMT program and acquisition of stemness by cancer cells is now supported by a substantial number of reports, although what discriminates these two processes remains largely elusive.

Here we show that overexpression of isoform δ of phosphoinositide 3-kinase (PI3Kδ) reprogrammed Huh7 cells to acquire a stem cell phenotype, forming a polarized rosette structure. This reprogramming was observed using AAV8-based PI3Kδ overexpression in the mouse liver. Notably, the pharmacological inhibition of PI3Kδ using CAL-101 promoted EMT. These PI3Kδ-mediated plasticity processes were dependent on TGFβ/SMAD7/Src and TGFβ/SMAD3 that control stemness and EMT respectively. Furthermore, the treatment of different HCC cells with CAL-101 induced morphological and functional differentiation. Taken together, our results suggest that PI3Kδ is a gatekeeper of the epithelium that controls plasticity. Modulation of its activity discriminates stemness from EMT, representing a step forward in understanding the development of aggressive carcinoma.

## Results

### PI3Kδ determines Huh7 cell derived-organoid morphogenesis

**Fig. 1:**
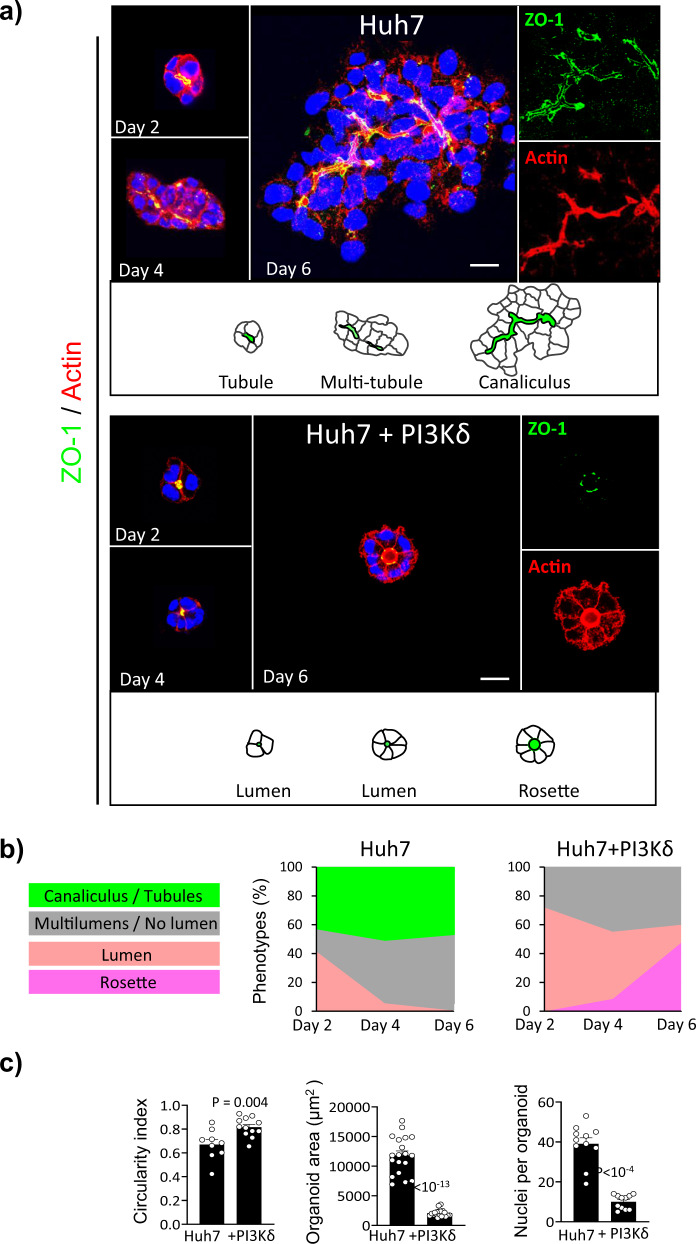
PI3Kδ is required for bile canaliculi formation and its overexpression induces formation of rosette-like structures. **a** Time-course analysis of lumen formation in Huh7 and Huh7 overexpressing PI3Kδ (Huh7 + PI3Kδ) organoids plated in 3D Matrigel-matrix and stained after 2, 4 or 6 days for Zonula-occludens 1 (ZO-1, green), actin microfilaments using phalloidin (red) and nuclei using Hoechst (blue). Scale bar: 10 μm. **b** Quantification of the phenotypes percentage over the days of culture of Huh7 (*n* = 117 organoids) and Huh7 + PI3Kδ (*n* = 123 organoids). **c** Quantification of Huh7 and Huh7 + PI3Kδ circularity index, organoids area and nuclei per organoid. Each dot of the graph corresponds to an organoid. All values are expressed as mean ± S.E.M.

To investigate the role of PI3Kδ in the liver, we used Huh7 cells derived from differentiated human HCC, representing the most studied cell culture system, to study liver physiopathogenesis, including infection by hepatitis C virus^21^. When cultured in 3D Matrigel, these cells organized to form actin-enriched tubules, delineated by apical Zona-Occludens 1 (ZO-1) staining, that were elongated and branched to form bile canaliculus-like structures on day 6 of culture (Fig. 1a, b). Interestingly, Huh7 cells overexpressing PI3Kδ (Huh7 + PI3Kδ) displayed a polarized rosette structure in which the cells were organized around a single central lumen (Fig. 1a, b, Supplementary Fig. 1a, b). The small size of the rosettes could be due to decreased cell proliferation, suggested by the decreased expression of several cyclin-dependent kinases (*CDKs*) and the increased expression of *P21*, an inhibitor of CDKs^22^ (Fig. 1c, Supplementary Fig. 1c). Notably, pharmacological inhibition of this enzyme in Huh7 + PI3Kδ cells impaired rosette formation, indicating that rosette formation was dependent on PI3Kδ activity (Supplementary Fig. 1d).

### PI3Kδ reprograms Huh7 cells into stem-like cells

**Fig. 2:**
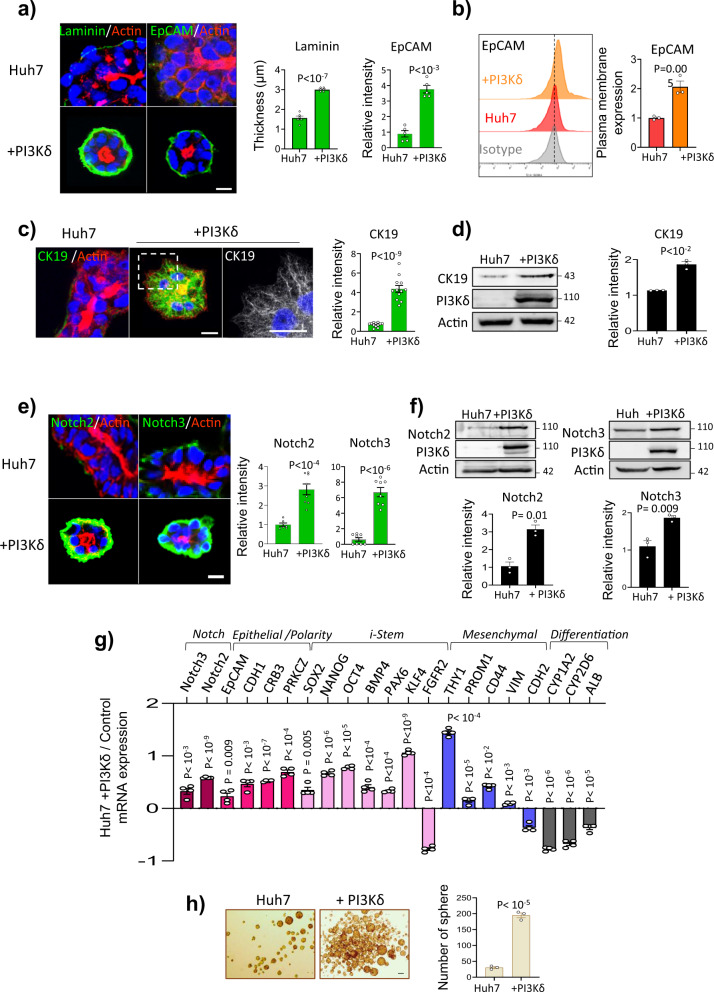
PI3Kδ activity regulates the plasticity of hepatocyte between epithelial and mesenchymal features. **a** Immunofluorescence staining in Huh7 and Huh7 + PI3Kδ after 6 days of 3D culture for Laminin-111 and EpCAM (green), actin microfilaments using phalloidin (red) and nuclei using Hoechst (blue). Scale bar: 10 μm. Quantification of laminin thickness and the relative intensity of EpCAM (right). Each dot of the graph corresponds to an organoid. **b** Flow cytometry analysis of EpCAM intensity at plasma membrane in Huh7 (red) versus Huh7 + PI3K cells 3 days after transfection (orange), control isotype is presented in gray. Right: Quantification of EpCAM mean intensity at the plasma membrane measured by flow cytometry analysis. **c** Immunofluorescence staining in Huh7 and Huh7 + PI3Kδ after 6 days of 3D culture for CK19 (green), actin microfilaments using phalloidin (red) and nuclei using Hoechst (blue). Scale bar: 10 μm. Quantification of the relative intensity of CK19 (right). Each dot of the graph corresponds to an organoid. **d** Immunoblot analysis of CK19 in Huh7 and Huh7 + PI3Kδ with the quantification of the relative intensity (*n* = 3 experiments). **e** Immunofluorescence staining in Huh7 and Huh7 + PI3Kδ after 6 days of 3D culture for Notch2 and Notch3 (green), actin microfilaments using phalloidin (red) and nuclei using Hoechst (blue). Scale bar: 10 μm. Quantification of the relative intensities (right). Each dot of the graph corresponds to an organoid. **f** Immunoblot analysis of Notch2 and Notch3 in Huh7 and Huh7 + PI3Kδ with the quantification of the relative intensity (below, *n* = 3 experiments). **g** RT-qPCR analysis of different genes performed in Huh7 and Huh7 + PI3Kδ organoids after 6 days of 3D culture. Data are presented as log_10_ mRNA fold change in Huh7 + PI3Kδ compared to Huh7 in two independent experiments performed in duplicate. **h** Spheroid formation assay in Huh7 and Huh7 + PI3Kδ, the number of spheres was determined after 5 days of culture based on two independent experiments performed in triplicate. All values are expressed as mean ± S.E.M.

This rosette-like structure consisting of Huh7 + PI3Kδ cells, surrounded by a dense ECM visible by laminin-111 labeling (Fig. 2a), is reminiscent of not only liver progenitor cells/small cholangiocytes^23^ but also stem cells that polarize during embryonic development^24^ and stem cells grown in 3D in Matrigel^25–27^. Thus, we analyzed the expression of epithelial surface protein (EpCAM) (Fig. 2a, b) and cytoskeleton protein cytokeratin 19 (CK19) (Fig. 2c, d), both of which are enriched in liver carcinomas with stem cell features, in the rosette cells^28,29^. We also investigated the Notch pathway, which plays a crucial role in the transdifferentiation and dedifferentiation of hepatocytes^30^. We observed increases in the Notch2 and Notch3 receptors in the Huh7 + PI3Kδ cells, indicating that PI3Kδ activates this pathway (Fig. 2e, f). Using RT-qPCR on organoids, we validated the increase of *Notch2* and *Notch3* gene expression (Fig. 2g). Furthermore, Huh7 + PI3Kδ cells expressed the key epithelial cell genes *EpCAM* and *CDH1*; and the polarity genes *CRB3* and *PRKCZ* (Fig. 2g), all of which are essential for the establishment and maintenance of apicobasal polarity^31,32^. The pluripotency genes *SOX2*, *OCT4* and *NANOG* and some of their upregulated targets genes like *BMP4, PAX6, KLF4* and *FGFR2*, a downregulated targets genes^33^; *VIM*, *CDH2* and the genes that encode the mesenchymal stem cell markers *CD44*, *CD90/THY1* and *CD133/PROM1*; also increased. We also observed decreased expression of the hepatocyte differentiation markers including the cytochrome P450 1A2 (*CYP1A2*) and 2D6 (*CYP2D6*) and albumin (*ALB*), suggesting a dedifferentiation process (Fig. 2g). In addition, the Huh7 + PI3Kδ cells formed more spheroids than control cells in the spheroid formation assay (Fig. 2h), reinforcing their stem cell characteristics. We validated PI3Kδ-induced Huh7 cell reprogramming in 2D culture using RT-qPCR and flow cytometry (Supplementary Fig. 2). Finally, we validated in HepG2 cells, which derived from a hepatoblastoma, a pediatric form of HCC, the role of PI3Kδ in inducing stemness (Supplementary Fig. 3). Thus, PI3Kδ reprogrammed Huh7 cells into stem-like cells with self-renewal capacity; the cells possessed the features of both mesenchymal and epithelial cells and exhibited columnar polarity when cultured in 3D in Matrigel.

### PI3Kδ is enriched in stem cells

We then performed a series of bioinformatics analyses to obtain more information about the potential involvement of PI3K in stemness. Using bioinformatics approaches based on GSE26093^34^, we showed high PI3Kδ expression in human embryonic stem cells (hESCs), induced pluripotent stem cells (iPSCs) and mesenchymal stem cells (MSCs) (Supplementary Fig. 4a). Furthermore, using GSE23034^35^, we analyzed the expression of PI3Kδ during the generation of iPSCs from mature human hepatocytes^35^. PI3Kδ expression significantly increased during the reprogramming process, while PI3Kα expression decreased, but no significant change in PI3Kβ or PI3Kγ was observed (Supplementary Fig. 4b). Subsequently, we hypothesized that PI3Kδ expression is important for embryonic development and needs to be downregulated to allow differentiation. To test this hypothesis, we analyzed the expression of PI3Kδ during the differentiation of hESCs into hepatocyte-like cells in vitro (GSE70741^36^). We validated the efficiency of the differentiation protocol described by the authors to generate hepatocyte-like cells and showed that they were capable of polarizing in Matrigel and forming bile canaliculi (Supplementary Fig. 5a, b). Then, using transcriptomics data from cells collected at different time points during hESC differentiation, we observed a gradual decrease in PI3Kδ expression, which was correlated with expression of the pluripotency factors nanog and oct4 and inversely correlated with expression of the differentiation genes albumin and HNF4α (Supplementary Fig. 5c, d). Thus, suggesting that PI3Kδ could have a role in development.

### PI3Kδ induced-reprogramming is dependent on TGFβ/Src

**Fig. 3:**
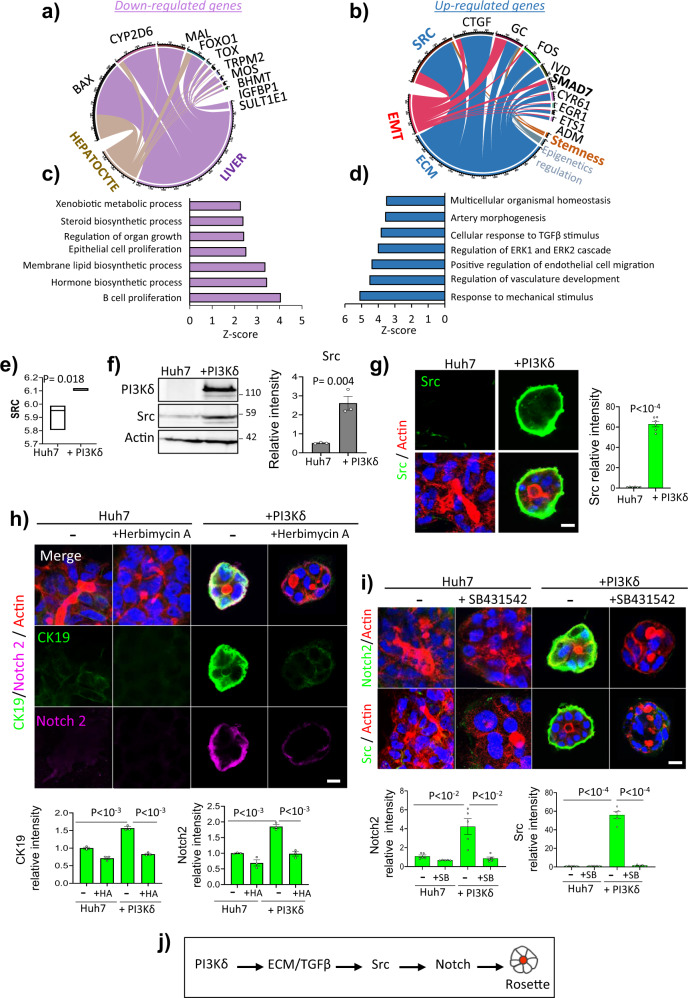
The PI3Kδ-induced phenotype in Huh7 cells is dependent on TGFβ/Src pathway. **a**, **b** Circosplot representing connection numbers during text-mining prioritization on pubmed of the genes downregulated (**a**) and upregulated (**b**) by PI3Kδ in transcriptomics analysis and terms hepatocyte, liver, EMT, ECM, epigenetics regulation and stemness. **c**, **d** functional enrichment of the gene downregulated (**c**) and upregulated (**d**) by PI3Kδ in transcriptomics analysis (GO-BP basis) **e** boxplot of Src expression in transcriptomics analysis. **f** Immunoblot analysis of Src in Huh7 and Huh7 + PI3Kδ with the quantification of the relative intensity (*n* = 3 experiments). **g** Immunofluorescence staining for Src (green), actin microfilaments using phalloidin (red) and nuclei (blue) in Huh7 and Huh7 + PI3Kδ organoids after 6 days of 3D culture. Scale bar: 10 µm. Quantification of the relative intensities (right). Each dot of the graph corresponds to an organoid. **h** Immunofluorescence staining for CK19 (purple), Notch2 (green), actin microfilaments using phalloidin (red) and nuclei (blue) in Huh7 and Huh7 + PI3Kδ organoids treated or not with the Src inhibitor herbimycin A at 100 nM during 6 days of 3D culture. Scale bar: 10 µm. Quantification of the relative intensities (below). Each dot of the graph corresponds to an organoid. **i** Immunofluorescence staining for Notch2 or Src (green), actin microfilaments using phalloidin (red) and nuclei (blue) in Huh7 and Huh7 + PI3Kδ organoids treated or not with 2 µM of the TGFβ inhibitor SB431542 during 6 days of 3D culture. Scale bar: 10 µm. Quantification of the relative intensities (below). Each dot of the graph corresponds to an organoid. **j** Proposed PI3Kδ dependant pathway for rosette-like structure formation in Huh7 expressing PI3Kδ. All values are expressed as mean ± S.E.M.

To gain further insight into the mechanisms involved in PI3Kδ-dependent reprogramming, we performed transcriptomic analysis of Huh7 + PI3Kδ cells versus control cells in triplicate, and a fold change in PI3Kδ of +53 was observed (Supplementary Fig. 6a). The Pavlidis template matching algorithm was applied, enabling the identification of 660 Affymetrix probes for genes co-regulated with PI3Kδ (312 positively regulated and 348 negatively regulated) (Supplementary data 1). Unsupervised classification of this expression profile enabled discrimination between the two conditions, as represented by a heatmap (Supplementary Fig. 6b) and confirmed by unsupervised principal component analysis (Supplementary Fig. 6c). Among the genes downregulated in Huh7 + PI3Kδ cells, *BAX*, *CYP2D6*, and *FOXO1* are involved in hepatocyte-specific processes such as xenobiotic metabolism and hormone and steroid synthesis (Fig. 3a, c), confirming that overexpression of PI3Kδ led to Huh7 cell dedifferentiation. Among the upregulated genes, most of them are related to ECM and thus reinforced the reported effect of PI3Kδ on ECM assembly in MDCK cells^12^ and the data from Fig. 2a. They included the non-receptor tyrosine kinase protein Src and connective tissue growth factor (CTGF) which is involved in most cellular responses to TGFβ and particularly those leading to ECM remodeling^37^. EMT and SMAD7 are also highlighted (Fig. 3b, Supplementary Fig. 7a). Interestingly, processes such as cellular response to TGFβ, extracellular signal-regulated kinase 1 (ERK1) and 2 (ERK2) cascade and the response to mechanical stimulus were highlighted in Huh7 + PI3K cells (Fig. 3d). The cues from mechanical stimuli arising from the ECM surrounding cells played important role in biological processes such as proliferation and differentiation^38^. Subsequently, we wondered if the effects of PI3Kδ on rosette formation were dependant of ECM signaling. First, we confirmed ERK, AKT and Src phosphorylation and the increase of Src (Fig. 3e–g, Supplementary Fig. 7b–d). We then investigated whether the PI3Kδ-induced phenotype depends on Src-mediated ECM signaling. Pharmacological inhibition of Src with herbimycin A impaired rosette formation and resulted in the formation of organoids that formed multiple lumens (Supplementary Fig. 7e). The expression of phosphorylated Src (^Y416^p-Src), Notch2 and CK19 decreased, as did the laminin surrounding the rosettes (Fig. 3h, Supplementary Fig. 7f and Supplementary Fig. 8a). Additionally, we assessed the involvement of TGFβ signaling in the PI3Kδ-induced rosette phenotype. Inhibition of the TGFβ receptor with SB431542 promoted the formation of organoids with multiple lumens and decreased Notch2 signal, similar to the effects of src inhibition (Fig. 3i). Interestingly, treatment with SB431542 strongly decreased p-SMAD2, vimentin and Src, indicating that TGFβ signaling regulated PI3Kδ-induced Src expression, which was required for the reprogramming of Huh7 cells into stem-like cells (Fig. 3j, Supplementary Fig. 8b).

### PI3Kδ reprograms hepatocytes in the mouse liver

**Fig. 4:**
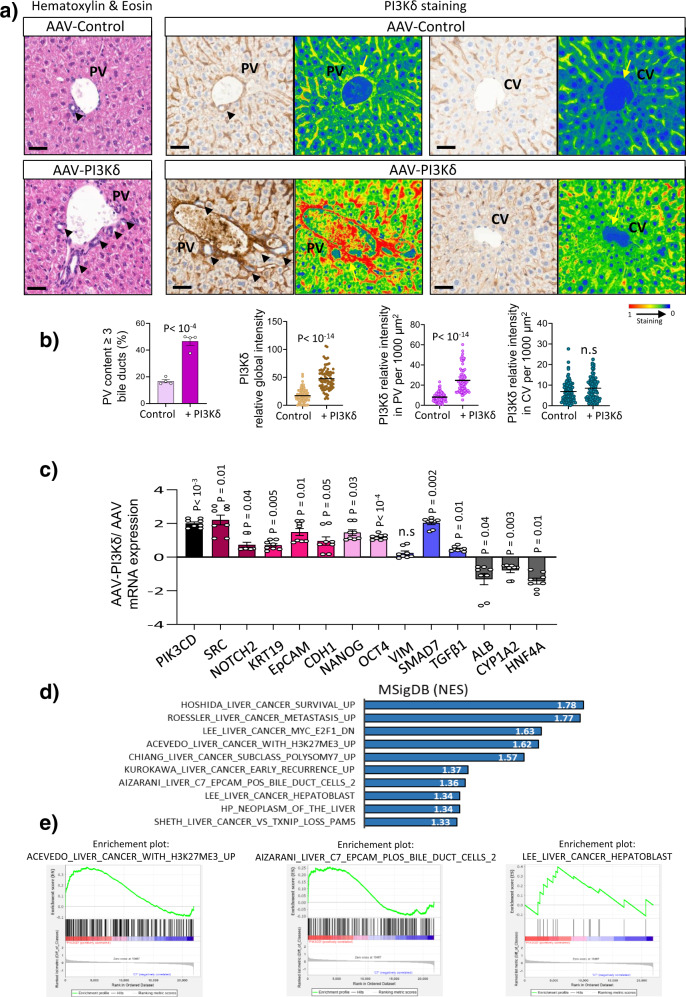
PI3Kδ overexpression in mouse liver induces histological changes and increase of stem cells markers. **a** Representative images of H&E staining of liver sections from mice injected with pAAV8-TBG-EGFP (AAV-control) (*n* = 4 mice) and with pAAV8- TGB- PI3Kδ (AAV-PI3Kδ) (*n* = 4 mice) allowing a visualization of liver architecture (portal vein (PV) and central vein (CV)) and ductular structure around the portal vein (PV). Scale bar: 50 µm. Right: Representative images of immunohistochemistry staining of PI3Kδ on AAV-control and AAV- PI3Kδ liver around the PV and CV structure. Colored images using case Viewer were presented at the right of each panel. Scale bar: 50 µm. **b** Bar graphs representing the percentage of portal vein (PV) with >3 small ductular structures. Bar graphs representing PI3Kδ global staining intensity and PI3Kδ staining intensity in PV and CV per 1000 µm^2^ (*n* = 20 for each mice). **c** RT-qPCR analysis of several markers expression indicated in the figure from AAV-PI3Kδ and AAV-control mice (duplicate measurement of *n* = 4 mice per condition). Data are presented as log_10_ mRNA fold change in AAV-PI3Kδ compared to AAV-control. **d** Liver signature of gene sets enriched in AAV-PI3Kδ. **e** Gene set enrichment analysis of upregulated genes in AAV-PI3Kδ. All values are expressed as mean ± S.E.M.

To study the relevance of our in vitro observations, we injected 8-week-old C57BL/6 mice with adeno-associated vector serotype 8 (AAV8), which has high affinity for mouse hepatocytes and has been suggested to transduce >90–95% of hepatocytes^39^*, via* intraportal vein injection. We used a plasmid encoding EGFP or mouse PI3Kδ under the liver-specific thyroid-binding globulin (TBG) promoter^40^ (pAAV TBG-EGFP (named AAV-control), pAAV TBG-PI3Kδ (named AAV-PI3Kδ)). Overexpression of PI3Kδ did not alter weight gain in the mice (Supplementary Fig. 9a). The infection of murine livers was verified by visualization of GFP fluorescence (Supplementary Fig. 9b). Hematoxylin and eosin staining revealed that PI3Kδ overexpression induced subtle ductular reaction characterized by numerous and disorganized small ductular structures around the portal vein (PV) (Fig. 4a, b) known to be associated with hepatocyte reprogramming^41,42^. PI3Kδ was assessed by immunohistochemistry and the signal was observed in sinusoids in line with its description in blood cells^43^. Nevertheless, in control mice a faint signal was observed around the PV (Fig. 4a, b); in AAV-PI3Kδ mice liver the PI3Kδ staining increased in all the liver and here again the signal was intense in sinusoids and around the PV. Although no major changes were observed at the central veins (Fig. 4a, b). Together, these data suggested an enhancement of PI3Kδ localization in the PV area of mouse liver. We thus performed RT-qPCR on the liver samples and data revealed the increase of epithelial and pluripotency genes, as well as the *Notch2, Src, TGFβ* and *Smad7* genes, associated with a decrease in expression of genes involved in hepatocyte differentiation were observed in AAV-PI3Kδ mouse livers (Fig. 4c).

Transcriptomic analysis revealed 73 upregulated genes in the livers of AAV-PI3Kδ mice, allowing discrimination of the two conditions (Supplementary data 2, Supplementary Fig 10a, b). Gene set enrichment analysis of the two conditions revealed that PI3Kδ regulates different cell functions, such as the response to hypoxia, apical junctions, mitotic spindle function and myogenesis (Supplementary Fig 10c–e). Gene set enrichment analysis of PI3Kδ-induced signatures revealed the enrichment of liver bipotency and stem cell phenotypes, as in liver cancer, which exhibits high levels of H3K27me3 marks^44^, EpCAM+ bile duct cells^45^, and hepatoblastoma, which exhibits the properties of hepatoblasts^46^ (Fig. 4d, e). Taken together, these results show that overexpression of PI3Kδ in the mouse liver induced the dedifferentiation of mouse hepatocytes, as observed in Huh7 + PI3Kδ cells (Fig. 2g). Thus, PI3Kδ overexpression reprograms hepatocytes into stem-like cells with polarity and epithelial features.

### PI3Kδ discriminates between EMT and stemness

**Fig. 5:**
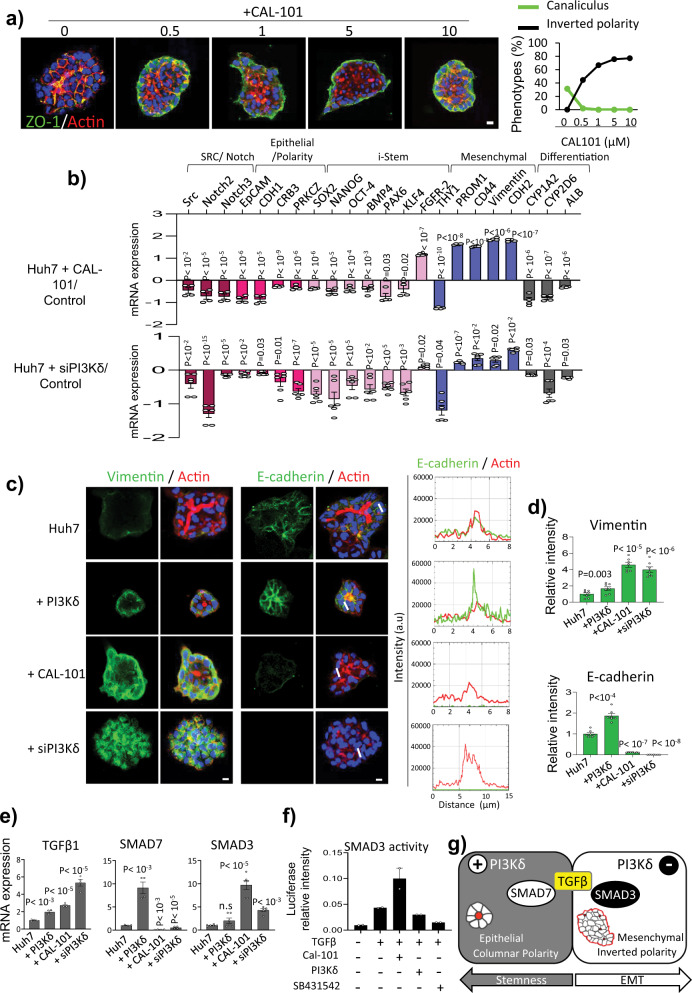
PI3Kδ activity discriminates EMT from stemness acquisition based on different TGFβ signaling. **a** Immunofluorescence staining for ZO-1 (green), actin microfilaments using phalloidin (red) and nuclei (blue) of 3D culture in organoids of Huh7 treated or not with PI3Kδ specific inhibitor idelalisib (CAL-101) at different concentrations after 6 days of 3D and quantification of the canaliculus and inverted polarity phenotype percentage in the different conditions (*n* > 49 for each condition). **b** RT-qPCR analysis of different genes expression performed in organoids of Huh7, Huh7+CAL-101 (5 µM) and Huh7 + siPI3Kδ after 6 days of 3D culture in two independent experiments performed in duplicate for Huh7 + 5CAL and in triplicate for HuH7 + siPI3Kδ. Data are presented as log_10_ mRNA fold change in Huh7+CAL-101 or Huh7 + siPI3Kδ compared to Huh7. **c** Immunofluorescence staining for the different markers indicated in the panel in organoids of Huh7, Huh7 + PI3Kδ, Huh7+CAL-101 (5 µM) and Huh7 + siPI3Kδ. Scale bar: 10 µm. The right panels present E-cadherin (green) and actin microfilaments using phalloidin (red) line profile blots of the white lines. **d** Quantification of relative intensity of these markers. Each dot of the graph corresponds to an organoid. **e** RT-qPCR analysis of different genes expression performed in organoids of HuH7, Huh7 + PI3Kδ, Huh7+CAL-101 (5 µM) and Huh7 + siPI3Kδ after 6 days of 3D culture in two independent experiments performed in duplicate. **f** Smad3 transcriptional activity, measured using CAGA-luciferase reporter, in HuH7, Huh7+ CAL-101 (5 µM) and Huh7 + PI3Kδ treated with TGFβ (2 ng/ml) and Huh7 treated with TGFβ and SB431542 (2 µM). Data represent a typical experiment performed in triplicate. **g** Schematic representation of the PI3Kδ-dependant plasticity with the EMT and stemness routes. All values are expressed as mean ± S.E.M.

To further investigate how PI3Kδ reprograms Huh7 cells, we studied the impact of PI3Kδ enzymatic activity. Treatment of Huh7 cells with idelalisib /CAL-101 at different doses altered the formation of canaliculi and resulted in the dose-response formation of organoids with an inverted polarity in which the apical domain stained by ZO-1 faced the ECM, whereas in control cells, ZO-1 stain the apical membrane facing the lumen of the tubules (Fig. 5a). This was reminiscent of our data from MDCK cells^12^. Subsequently, we analyzed the expression of several genes, as done upon PI3Kδ overexpression (Fig. 2g). Overall, PI3Kδ inhibition using 5 µM CAL-101 or siPI3Kδ significantly decreased *Src* and *NOTCH2/3* genes, epithelial genes and pluripotency factors and their target genes as expected. Strikingly, mesenchymal markers were significantly increased, and the effect was more pronounced than that upon PI3Kδ overexpression. Here, again, these changes were associated with the loss of hepatocyte markers (Fig. 5b). Similar data were observed at different concentration of CAL-101 (Supplementary Fig. 10a–c), suggesting a dedifferentiation and EMT processes with a decrease of Src and p-AKT level (Supplementary Fig. 11d, e). Comparing the control and siPI3Kδ condition in RT-qPCR, we didn’t detect significant changes regarding the other isoforms of class I PI3Ks, confirming that the effects seen is due to the δ isoform (Supplementary Fig. 11g). We also confirmed the changes in *CDH1, VIM* and *EpCAM* gene expression by immuofluorescence analyses of the encoded proteins (Fig. 5c, d, Supplementary Fig. 11f). Notably, the localization of E-cadherin at cell–cell contacts reinforced the notion that Huh7 + PI3Kδ cells are highly polarized epithelial cells (Fig. 5c). Therefore, PI3Kδ inhibition induced epithelial to mesenchymal transition (EMT), characterized by decreased epithelial gene expression and increased mesenchymal marker expression, consistent with the inverted polarity phenotype of the organoids (Fig. 5a). Indeed, EMT has been shown to reverse the apicobasal polarity axis^47^.

### Different TGF β signaling pathways control EMT and stemness

TGFβ is a master regulator of the EMT process^48^. We therefore investigated whether TGFβ signaling was also involved in the polarity inversion of organoids generated upon PI3Kδ inhibition. The addition of SB431542 to inverted polarized organoids restored the canaliculi-like structure of the Huh7 organoids, and the cells no longer expressed vimentin (Supplementary Fig. 11h). We therefore sought to validate these observations in MDCK cells, a well-established model for the study of epithelial cell plasticity. As in Huh7 cells, inhibition of PI3Kδ induced polarity inversion and the increase of N-cadherin in MDCK organoids (Supplementary Fig. 12a), whereas PI3Kδ overexpression increased src signal both in a TGFβ signaling-dependent manner (Supplementary Fig. 12a–c). Interestingly RT-qPCR data confirmed the changes in induced pluripotency factors, polarity gene *PRKCZ*, the transcripts for E-cadherin, vimentin and N-cadherin **(**Supplementary Fig. 12d). TGFβ signaling controls plasticity and was involved here in the formation of both the rosette structure and inverted polarity organoids; thus, it contributes to EMT and stemness. To distinguish these processes, we studied signaling downstream of TGFβ receptors upon the modulation of PI3Kδ activity. Mothers against decapentaplegic homolog (SMADs) are the main transducers of TGFβ receptor signaling. These proteins are notably involved in the regulation of EMT induced by TGFβ. While SMAD3 is one of the main effectors of TGFβ-induced EMT^49^, SMAD7 is a known inhibitor of this process^50^ and was recently found to promote stemness^51^. Strikingly, *SMAD3* was upregulated in Huh7+CAL-101 and Huh7 + siPI3Kδ cells (Fig. 5e), whereas *SMAD7* was upregulated in Huh7 + PI3Kδ cells (Figs. 3b, 5e) and downregulated in Huh7+CAL-101 cells (Fig. 5e). We also measured SMAD3 activity following the addition of TGFβ using the luciferase reporter CAGA^52^. We observed enhanced TGFβ-induced SMAD3 activity in Huh7+CAL-101 cells and conversely, the stimulation of Huh7 + PI3Kδ cells with TGFβ decreased SMAD3 activity (Fig. 5f). Overall, these data reveal that PI3Kδ activity regulates different TGFβ-dependent pathways, ultimately leading to different cellular responses, and highlights the differences between stemness and EMT, as illustrated in Fig. 5g.

### PI3Kδ activity controls plasticity and the fate of different HCC cells

**Fig. 6:**
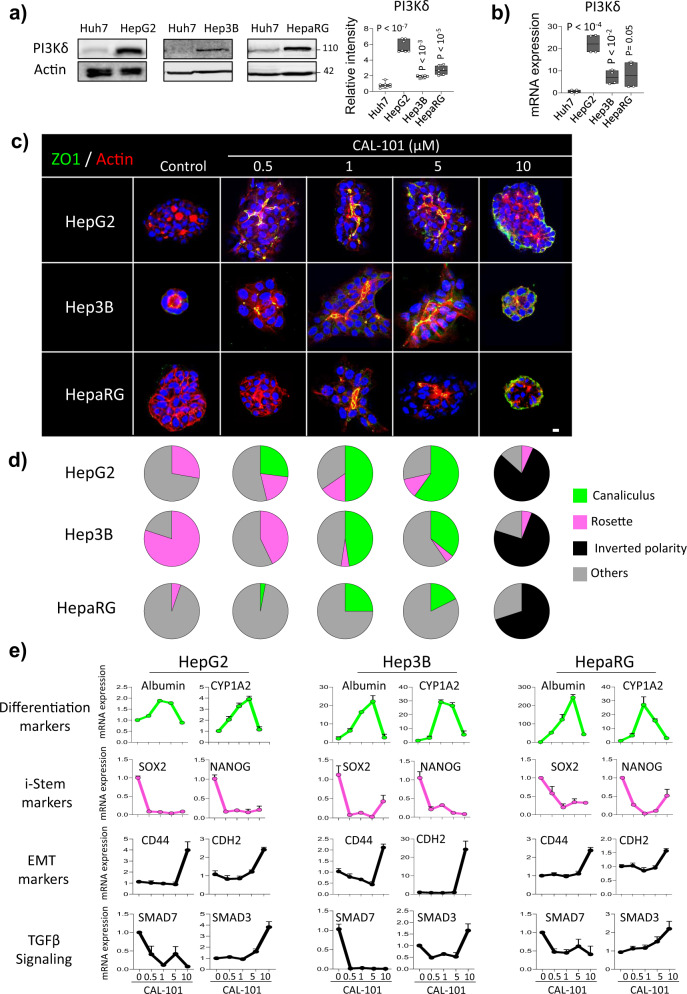
Inhibition of PI3Kδ activity improves canaliculus formation and differentiation in HepG2, Hep3B and HepaRG cells associated with the regulation of EMT and stemness markers. **a** Immunoblot analysis of PI3Kδ protein level in Huh7, HepG2, Hep3B and HepaRG with the quantification of its relative intensity (right, *n* = 3 experiments). **b** RT-qPCR analysis of PI3Kδ expression in Huh7, HepG2, Hep3B and HepaRG cells; RPLP0 was used as the housekeeping gene for normalization. **c** Immunofluorescence staining for ZO-1 (green), actin microfilaments using phalloidin (red) and nuclei (blue) in HepG2, Hep3B and HepaRG cells plated in 3D cultures for 6 days and treated with the PI3Kδ specific inhibitor (CAL-101) at different doses. Scale bar: 10 µm **d** Quantification of the percentage of the different phenotypes seen in the conditions above (*n* = 40 organoids). **e** RT-qPCR analysis of several markers expression in HepG2, Hep3B and HepaRG cells plated in 3D culture and treated with different doses of CAL-101 for 6 days in two independent experiments performed in duplicate; RPLP0 was used as the housekeeping gene for normalization. All values are expressed as mean ± S.E.M.

**Fig. 7:**
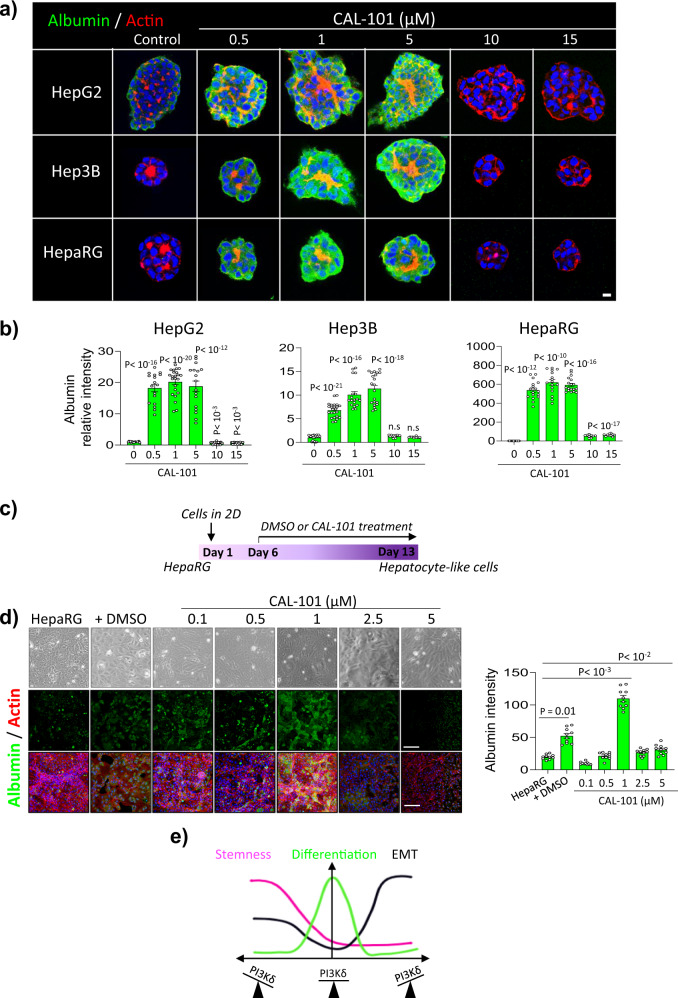
The inhibition of PI3Kδ activity increases albumin protein level in different hepatic cell lines. **a** Immunofluorescence staining for Albumin (green), actin microfilaments using phalloidin (red) and nuclei (blue) in HepG2, Hep3B and HepaRG cells plated in 3D cultures for 6 days and treated or not with the PI3Kδ specific inhibitor (CAL-101) at different doses. Scale bar: 10 µm. **b** Quantification of albumin relative intensity in the different cell lines treated or not with CAL-101 at different doses. **c** Experiment plan for HepaRG differentiation using DMSO or CAL-101. **d** Immunofluorescence staining for Albumin (green), actin microfilaments using phalloidin (red) and nuclei (blue) in HepaRG cells treated with DMSO or CAL-101 at different doses after 7 days of treatment. Scale bar: 100 µm. Quantification of albumin relative intensity in HepaRG within the different conditions above. **e** Proposed PI3Kδ dependant pathway discriminating EMT from stemness. All values are expressed as mean ± S.E.M.

In order to gain further insights regarding the role of PI3Kδ in liver cell polarization and differentiation, we used CAL-101 to treat HCC cells with high expression level of PI3Kδ including HepG2, Hep3B, another hepatoblastoma derived cell lines and the hepatic bi-progenitor cell line HepaRG (Fig. 6a, b). Unlike Huh7 cells, these cells were not able to form bile canaliculi on 3D culture after 6 days rather HepG2 and Hep3B could form rosette structure (Supplementary Fig. 13). We then inhibited PI3Kδ activity in those cells with increasing doses of CAL-101. Surprisingly, we observed a rescue of bile canaliculi formation with a maximum effect at 1 and 5 µM for all the cell lines (Fig. 6c, d). Interestingly, the presence of canaliculi in the different cell lines was associated with the increase of the expression of differentiated hepatocyte markers such as albumin and the cytochrome P450 1A2 (*CYP1A2*), cytochrome P450 2D6 (*CYP2D6*) (Fig. 6e and supplementary Fig. 14a, b). Concomitantly, the induced pluripotent transcription factors *Nanog* and *SOX2* significant decreased in a dose dependent manner while *CD44* and *CDH2* used as EMT markers were very low and abruptly increased significantly at 10 µM where organoids adopted inverted polarity phenotype with no canaliculi formation (Fig. 6d, e). Interestingly, we also observed a decrease of *SMAD7* transcripts while we detected a significantly increases at 10 µM of CAL-101 for *SMAD3* (Fig. 6e). All together, these data strongly suggested that high PI3Kδ activity controlled stemness and its modulation induced hepatocytes cell differentiation as validated by immunofluorescence staining of albumin (Fig. 7a, b) and its full inhibition promoted EMT (Fig. 6e). These data also provided evidence that these plastic events are regulated by different TGFβ-dependent pathways and highlighting the differences between stemness and EMT in different hepatic cell lines.

Finally, we performed a well described method for 2D culture by which HepaRG cells were able to acquire the differentiated hepatocyte phenotype when treated with DMSO^53^ (Fig. 7c). HepaRG cells were grown to confluence for 7 days and treated with 1.8%DMSO or different concentrations of CAL-101 for 7 days. We observed a more than two folds increase of albumin staining in HepaRG + 1 µM CAL-101 comparing to HepaRG +DMSO (Fig. 7c, d). These data provide a distinct role of PI3Kδ in hepatocyte differentiation and may serve as a new tool to acquire differentiated hepatocyte in vitro and both in 2D and in 3D.

## Discussion

In this study and as summarized in Fig. 7e, we showed that PI3Kδ controls plasticity in epithelial cells and that its balance is required to maintain epithelial cell polarity and differentiation. In the liver, this results in the formation of bile canaliculi and the functional differentiation of hepatocytes. Inhibition of PI3Kδ induced EMT, revealed by an increase in mesenchymal genes and a decrease in epithelial and polarity genes in cells that formed inverted polarized organoids. Conversely, PI3Kδ overexpression in cells promoted stemness, characterized by an increase in both mesenchymal and epithelial markers and pluripotency factors.

To note that the stemness was described as a broad window on the halfway to EMT, a stage named hybrid EMT^54–56^. Here, we propose that distinct routes promote the stemness which represents a gain of epithelial and pluripotency factors, while EMT, as it is largely defined, is accompanied by the loss of these genes^57^. Importantly, PI3Kδ activity allows the discrimination of these two processes, both of which require TGFβ-dependent plasticity. We identified SMAD3 and SMAD7 as the main downstream effectors of TGFβ that control the divergence between EMT and stemness, respectively.

Interestingly, our data also showed that PI3Kδ expression was elevated in different stem cells, including hESCs, thus strongly supporting its role in the developmental stemness. Furthermore, the profile of each of the class I PI3K isoforms appeared different and indeed, the PI3Kδ expression was elevated in the stem cells and was found correlated to pluripotency factors expressions. By contrast the PI3Kα expression decreased in stem cells and its expression increased along with differentiation process. Thus, it will be important to decipher the spatio-temporal regulation of stemness and plasticity by PI3Kδ and the contribution of other isoforms which remained enigmatic^58^. The crucial role of signaling pathways such as Notch in liver plasticity has been reported^30^. Interestingly, we were able to demonstrate in our study that the Notch pathway is activated by PI3Kδ.

Moreover, we studied the effects of CAL-101 treatment on different HCC-derived cells grown on 3D and data showed different scenarios regarding drug concentration which are also related to PI3Kδ expression. Indeed, using Huh7 with low expression of PI3Kδ, treatment induced loss of differentiation and promoted EMT. However, cells such as HepG2, Hep3B and HepaRG which have a high expression of PI3Kδ are able to form canaliculi and expressed hepatocyte differentiation markers. However, at the highest dose of CAL-101 formed inverted polarized structures and with EMT features. Thus PI3Kδ appeared as a central regulator of epithelial cells plasticity in liver as well as in MDCK cells. Furthermore, the establishment of relevant in vitro culture systems is a challenge for the toxicology assessment of drugs by the pharmaceutical industry and for the study of liver cell biology. In this context, CAL-101 appeared here as an efficient tool for in vitro hepatic differentiation.

We noticed that PI3Kδ overexpression creates a dense layer of ECM around the rosettes as previously described with MDCK cysts^12^ and furthermore the bioinformatic data (Fig. 3b) highlighted an increase of ECM upon PI3Kδ overexpression. Therefore, we demonstrated here that Src is an important regulator of the signaling from the cell-ECM interactions required for PI3Kδ-dependent morphogenetic effects. ECM plays important role during differentiation of liver cells as well as in carcinoma development. Furthermore, nearby 80% of HCC are established on cirrhotic liver presenting ECM alteration. Together, these findings open new perspectives for investigating the role of PI3K isoforms in the plasticity of epithelial cells in both development and cancer conditions which remained open questions^58^.

## Methods

### Cells and 3D culture

Huh7, Hep3B and HepG2 cell lines (from ATCC) were cultured in Dulbecco’s modified Eagle’s Medium containing 4.5 g/L glucose supplemented with 10% heat-inactivated fetal bovine serum, 1% non-essential amino acids, 1% sodium pyruvate and 1% of penicillin/streptomycin at 37 °C in 5% CO. HepaRG cell line (from Biopredic) was cultured in William’s E medium supplemented with 10% fetal bovine serum, 1% penicillin/streptomycin, 5 µg/mL insulin and 5 × 10^−5^ M hydrocortisone hemisuccinate. To obtain HepaRG differentiation, cells were cultured in the same medium as above for 2 weeks supplemented with 1.8% dimethylsulfoxide (DMSO). MDCK (Madin Darby Canine Kidney from Keith Mostov, UCSF, San Francisco) cells were cultured in minimal essential medium (MEM) supplemented with 5% fetal bovine serum and 1% of penicillin/streptomycin. For the 3D culture, cells were trypsinized as 10,000 cells/mL in 2% Matrigel (BD Biosciences). 500 µL of cells were plated in each well of eight-well Lab-Tek II chamber slides (Thermo Fisher Scientific) coated with Matrigel and grown for up to 6 days.

### Human embryonic stem cells maintenance, differentiation and 3D culture

Undifferentiated human H1 ES cells (WiCell) were maintained in monolayer culture on Matrigel (BD Biosciences) in mTeSR1 medium (Stemcell Technologies, 05850) at 37 °C with 5% CO2. Cells were manually passaged at 1:4 to 1:6 split ratios every 3–5 days. For hepatic differentiation, cells were cultured for 3 days in RPMI/B27 medium (Insulin minus, Gibco, A18956-01) supplemented with 100 ng/ml Activin A (Peprotech, 120-14E), followed by 4 days with 20 ng/mL BMP2 (Peprotech, 120-02) and 30 ng/mL FGF-4 (Peprotech, 100-31) in RPMI/B27 (complete with Insulin, Gibco, 17504-044) medium, then 6 days with 20 ng/mL HGF (Peprotech, 100-39) and KGF (Peprotech, 100-19) in RPMI/B27 (complete with Insulin), then 8 days with 20 ng/mL Oncostatin-M (R&D Systems, 295-OM/CF) in hepatocyte culture media (Lonza, cc-3198) supplemented with SingleQuots (without EGF). All cell lines used were negative for mycoplasma contamination. For 3D culture, cells were dissociated as small patch at day 16 using accutase and manual pipetting and add to a solution of 40% matrigel containing hepatocyte culture medium supplemented with SingleQuots (without EGF). Then cells were placed as drop in eight-well Lab-Tek II chamber slides and culture for 6 days before fixation.

### Plasmids, siRNA and cell transfection

Human p110δ cDNAs were obtained from Bart Vanhaesebroeck, University College London. The specific p110δ duplex RNAi used were: si3: 5′-CAGAUGAGAAGGGCGAGCUGCUGAA-3′ and 5′-UUCAGCAGCUCGCCCUUCUCAUCUG-3′. For transfection, the cells were seeded at the density of 1.10^5^ cells/well of a 12-well-plate and transfected with 100 pmol of specific siRNA or 2 µg cDNA using jetPRIME (Ozyme), according to the manufacturer’s instructions. For 3D culture, 24 h after the transfection, cells were detached using trypsin and plated on Matrigel as indicated above.

### Immunoblot

Cells were lysed in Laemmli sample buffer and denatured at 100 °C for 5 min before separation on 10% SDS–PAGE and then electrotransferred onto nitrocellulose blotting membrane (Amersham Protran). After transfer, the membrane was saturated in DPBS containing 0.1% Tween 20 and 5% milk. Primary antibodies (appropriate dilution) were added overnight at 4 °C. After washes in the presence of DPBS, appropriate secondary antibodies coupled with peroxidase were added. Immunoblotting was revealed with chemiluminescent peroxidase substrate (Chemiluminescent Peroxidase Substrate-3; Sigma-Aldrich) and exposure was observed with G: box (Syngene).

### Immunofluorescence staining

The cells were rinsed with ice-cold Dulbecco’s PBS (DPBS) and fixed with 4% paraformaldehyde for 20 min at 4 °C. The samples were then permeabilized and saturated with DPBS supplemented with 0.7% fish gelatin and 0.025% saponin for 30 min at 37 °C before being incubated with primary antibodies. After washing, staining was performed with secondary fluorescent antibodies, phalloidin for F-actin and Hoechst-33342 for nuclei. Images were acquired using a Leica TCS SP5 AOBS tandem 30 confocal microscope and presented as a single confocal section through the middle of the cyst. Images were analyzed using ImageJ.

### Antibodies and chemicals

Two primary antibodies were used for p110δ: the rabbit anti p110d from Santa Cruz sc7176 for immunoblot (1:1000 dilution) and the anti-PI3- Kinase p110d antibody from Abcam ab32401 (1:100 dilution used for immunofluorescence). The other antibodies used are detailed in Supplementary data 3.

Idelalisib was purchased from Selleckchem, SB-431542 from Invivogen, recombinant human TGF-β1 and herbimycin A from ChemCruz.

### Quantitative reverse transcription–PCR

Total RNA was isolated using RNeasy Mini Kit 50 (Qiagen) and applied to reverse transcription using the RevertAid First Strand cDNA Synthesis Kit (Thermo Fisher). The cDNA was analyzed by qPCR using the LightCycler 480 SYBR Green I Master (Roche) with a LightCycler^®^ 96 Instrument (Lifescience, Roche). The reaction parameters were 50 °C for 30 min, 95 °C for 15 min, followed by 40 cycles of 94 °C for 30 s, 55 °C for 30 s and 72 °C for 30 s. The triplicate mean values were calculated using RPLP0 gene transcription as the reference for normalization. The primers used are indicated in Supplementary data 4.

### Spheroid formation assay

Huh7 cells transfected or not with PI3Kδ expression vector were seeded as 500 cells/well in a 24-well ultra-low attachment plates with lid flat bottom (Corning).The number of spheres was counted using a microscope from triplicate experiments after 5 days of culture.

### Flow cytometry

Cells were dissociated using Accutase followed by a neutralization step with culture media. 1/100 of primary antibody was added for 1 h, and after washing the cells were stained and incubated with the secondary antibody for 1 h. Fluorescence intensity was measured by flow cytometry with BD Accuri C6 plus software. Data Analysis was performed using the FlowJo software.

### CAGA-Luciferase reporter experiments

Cells were co-transfected with vectors coding for the gene of interest with CAGA-Luc reporter plasmids and the Renilla luciferase plasmid to normalize the results. Cells were incubated 24 h later in the presence or absence of TGF-β1 and with or without CAL-101 for another 18 h. Luciferase activity was measured with the Dual-Luciferase Reporter Assay (Promega) system according to the manufacturer’s instructions.

### RNA preparation and transcriptome analysis

Total RNA from the Huh7 cells was prepared using the RNeasy Mini Kit 50 (Qiagen) or using Trizol (Invitrogen) and following the manufacturer’s recommendations. RNA was quantified using Nanodrop technology and the quality of nucleic acid was verified using a Bioanalyser (Agilent Technologies). Triplicate total RNA samples which passed quality controls was used to synthetize an amplified RNA (aRNA) microarray probe using the linear T7 RNA polymerase amplification protocol (Affymetrix). Labeled an RNA probes were hybridized on the human Affymetrix Microarray ST2.0. The microarray was then scanned by the Affymetrix platform and normalized using the RMA algorithm included in the Affymetrix expression console. The RNA-Seq data discussed in this publication have been deposited with GEO under the accession number GSE128022.

### Bioinformatics analysis of the transcriptome

A bioinformatics analysis was performed in the R software environment (version 3.0.2). An RMA normalized matrix of the Huh7 transcriptome was used with the R Bioconductor genefilter package in order to remove invariable genes. The Pavlidis template matching algorithm was used to determine genes co-regulated to PIK3CD which was transfected in Huh7 cell: the threshold of correlation was fixed at *R* ≥ 0.80 in absolute value. Microarray expression heatmaps were produced using the MADE4 R-package. Unsupervised principal component analysis on the gene expression matrix was performed with the FactoMineR R-package. Functional enrichment on the gene ontology biological process for microarray analysis was performed using Enrichr website tools.

### AAV-2/8 vectors production for PI3Kδ expression in vivo

AAV gene transfer plasmids were purchased at Vector Builder: pAAV TBG m PI3Kδ (VB180205-1022wdw) allowing mouse PI3Kδ CDS expression under the control of TBG promoter (liver-specific promoter) and pAAV TBG-EGFP [VB180202-1128wha]. These plasmids were first amplified and then co-transfected into 293AAV Cell Line (Cell Biolabs, AAV-100) together with a helper plasmid (Part No. 340202 VPK-401 kit) and REP-Cap plasmid (pAR-(rh)8, a kind gift of Dr. Miguel Esteves (Gene Therapy Center, University of Massachusetts Medical School, 368 Plantation Street, ASC6-2055, Worcester, MA 01605)). After 3 days, cells were lysated and rAAV were collected and clarified. rAAV vectors were then titrated with RT-qPCR Adeno-Associated Virus Titration (Titer) Kit (ABMGood, G931) and used for in vivo injections (1E11 TU/mouse).

### Mice and lentiviral transduction

C57BL/6 mice (purchased from Charles River) were maintained under specific pathogen-free conditions, and food and water were provided ad libitum. Mice were infected at 8 weeks old using pAAV TBG m PI3Kδ and pAAV TBG-EGFP adenovirus (10^11^ particules / mouse) and sacrificed at 12 weeks old (sex: male). C57BL/6 mice purchased from Charles River were maintained under specific pathogen-free conditions, and food and water were provided ad libitum. Mice were infected in the tail vein at 8 weeks after birth using pAAV TBG m PI3Kδ and pAAV TBG-EGFP adenovirus (10^11^ particules/mouse) and sacrificed at 12 weeks after birth. Mice were bled under anesthesia via retro-orbital plexus and sacrificed by cervical dislocation. One lobe of the liver was divided in three parts: one lobe was fixed in 4% formalin for 24 h and embedded in paraffin, one lobe was placed in Tissue-Tek OCT compound and stored at −80 °C and the rest of the liver was snap frozen and kept at −80 °C. The sample in paraffin was used for immunohistochemistry staining (using case viewer and QuPath software) and the frozen tissue was used for RNA extraction and RT-qPCR analyses. The University of Liege ethical committee approved all protocols under number 1738.

### Liver mice transcriptome

With total RNA from tumors transcriptome Clariom S mouse was performed for three individual samples in each experimental condition: transfection with empty vector and transfection with PIK3CD vector. Microarray were normalized Robust Microarray Analysis (RMA) method^59^. Rank products analysis with False Discovery Rate correction between sample conditions was performed to identify genes regulated between experimental conditions^60^. Gene set enrichment analysis was performed between sample conditions^61^ and functional enriched networks were built with Cytoscape version 3.6.0^62^. Bioinformatics analysis were followed in R software environment version 4.0.2 with some packages: pheatmap for drawing expression heatmap and FactoMineR for principal component analysis.

### Statistics and reproducibility

All values are expressed as mean±S.E.M. Comparisons of mean values were performed using an unpaired Student’s *t*-test on 3 independent experiments excepted when mention in legend.

### Reporting summary

Further information on research design is available in the Nature Research Reporting Summary linked to this article.

## Supplementary information

Supplementary Information

Description of Additional Supplementary Files

Supplementary Data 1

Supplementary Data 2

Supplementary Data 3

Supplementary Data 4

Supplementary Data 5

Reporting Summary

## Data Availability

Raw data from affymetrix microarray have been deposited under the following code: GSE128022 for the transcriptomes of Huh7 and Huh7 + PI3Kδ. GSE183392 for the transcriptomes of AAV and AAV-PI3Kδ. All the original full blots for the cropped images are shown in the supplementary Fig. 15-18. Source data behind the graphs are available in Supplementary Data 5.
