# Supplementary material for: PI3Kδ activity controls plasticity and discriminates between EMT and stemness based on distinct TGFβ signaling
Source: Commun Biol. 2022 Jul 25;5:740. doi: 10.1038/s42003-022-03637-w (PMC9314410; doi:10.1038/s42003-022-03637-w)
Supplement: Supplementary file 1 — Supplementary Information [file 42003_2022_3637_MOESM1_ESM.pdf]

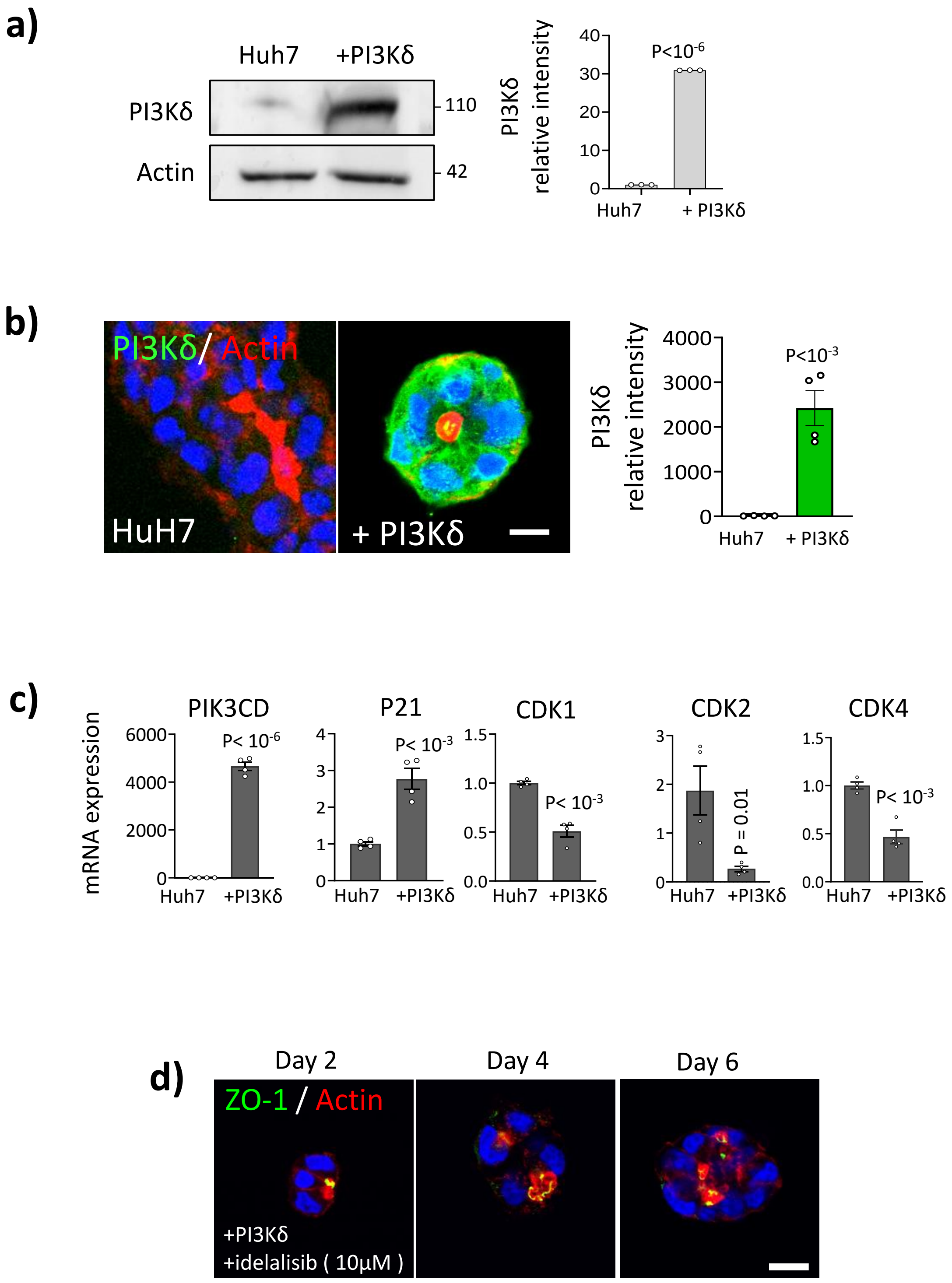
a)
Huh7
+PI3Kδ
PI3Kδ
Actin
110
42
PI3Kδ relative intensity
P<10-6
Huh7
+ PI3Kδ
b)
PI3Kδ/ Actin
HuH7
+ PI3Kδ
PI3Kδ relative intensity
P<10-3
Huh7
+ PI3Kδ
c)
mRNA expression
PIK3CD
P< 10-6
P21
P< 10-3
CDK1
P< 10-3
CDK2
P = 0.01
CDK4
P< 10-3
Huh7
+PI3Kδ
d)
Day 2
Day 4
Day 6
ZO-1 / Actin
+PI3Kδ
+idelalisib (10μM)

Supplementary Figure 1. PI3Kδ induces of rosette-like structures is associated with a decrease of cell proliferation markers and is dependant on its kinase activity

a) Immunoblot analysis of PI3Kδ in Huh7 control and Huh7+PI3Kδ with the quantification of its relative intensity (right, n= 3 experiments). **b)** Immunofluorescence staining for PI3Kδ (green), actin microfilaments using phalloidin (red) and nuclei (blue) in Huh7 control and Huh7+PI3Kδ organoids after 6 days of 3D culture. Scale bar: 10µm. Quantification of the relative intensity (right). Each dot of the graph corresponds to an organoid. **c)** RT-qPCR analysis of different genes expression performed in Huh7 and Huh7+PI3Kδ organoids after 6 days of 3D culture in two independent experiments performed in duplicate. RPLP0 was used as the housekeeping gene for normalization. **d)** Time-course analysis of lumen formation in Huh7+PI3Kδ organoids treated or not with 10µM of CAL-101 and stained for Zonula-occludens 1 (ZO-1) antibody (green), actin microfilaments using phalloidin (red) and nuclei using Hoechst (blue) after 2, 4 or 6 days of 3D culture. Scale bar: 10 µm. All values are expressed as mean±S.E.M.

a)

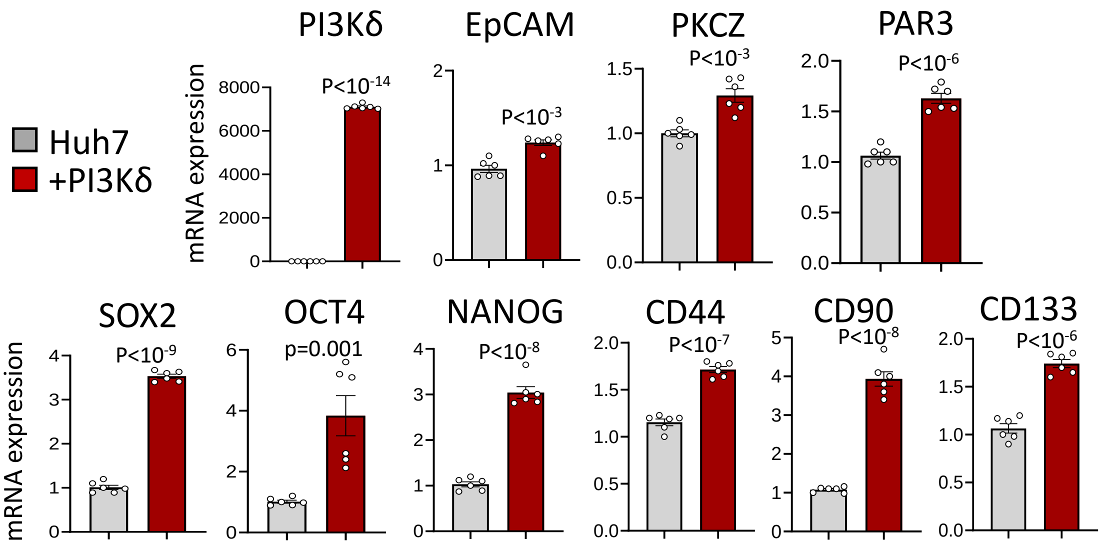

PI3Kδ
EpCAM
PKCZ
PAR3
Huh7
+PI3Kδ
mRNA expression
P<10-14
P<10-3
P<10-3
P<10-6
SOX2
OCT4
NANOG
CD44
CD90
CD133
P<10-9
p=0.001
P<10-8
P<10-7
P<10-8
P<10-6

b)

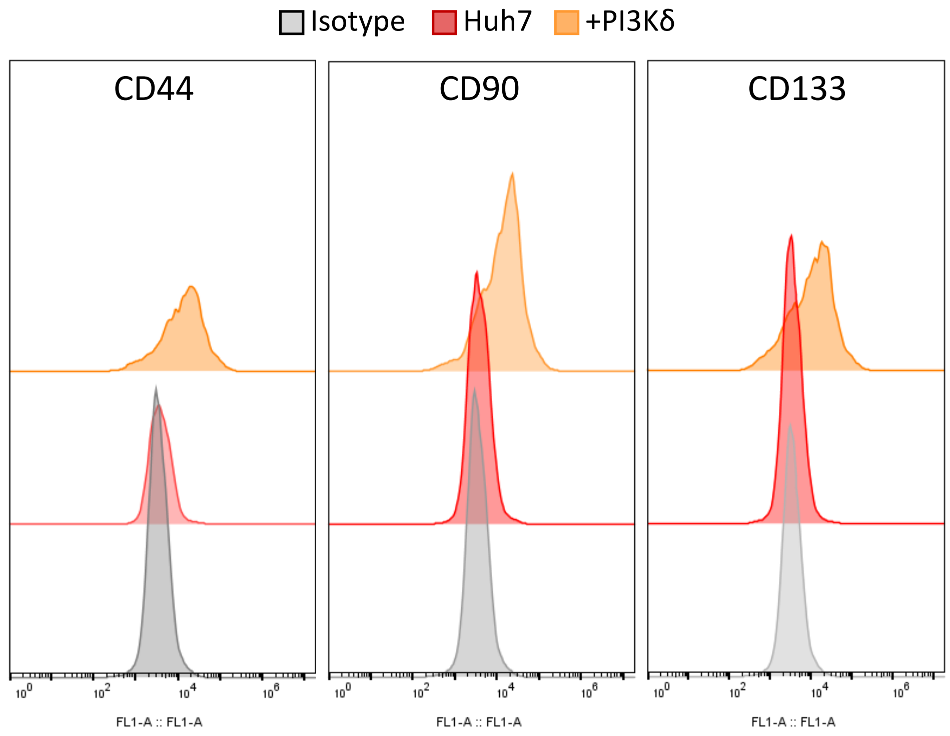

Isotype
Huh7
+PI3Kδ
CD44
CD90
CD133
FL1-A :: FL1-A
FL1-A :: FL1-A
FL1-A :: FL1-A

Supplementary Figure 2. PI3Kδ induced an activation of progenitors markers

a) RT-qPCR analysis of different genes expression performed in Huh7 and Huh7+PI3Kδ (n = 2 independent experiments performed in triplicate). **b)** Flow cytometry analysis of CD44, CD90 and CD133 at plasma membrane in Huh7 and Huh7+PI3Kδ. All values are expressed as mean±S.E.M.

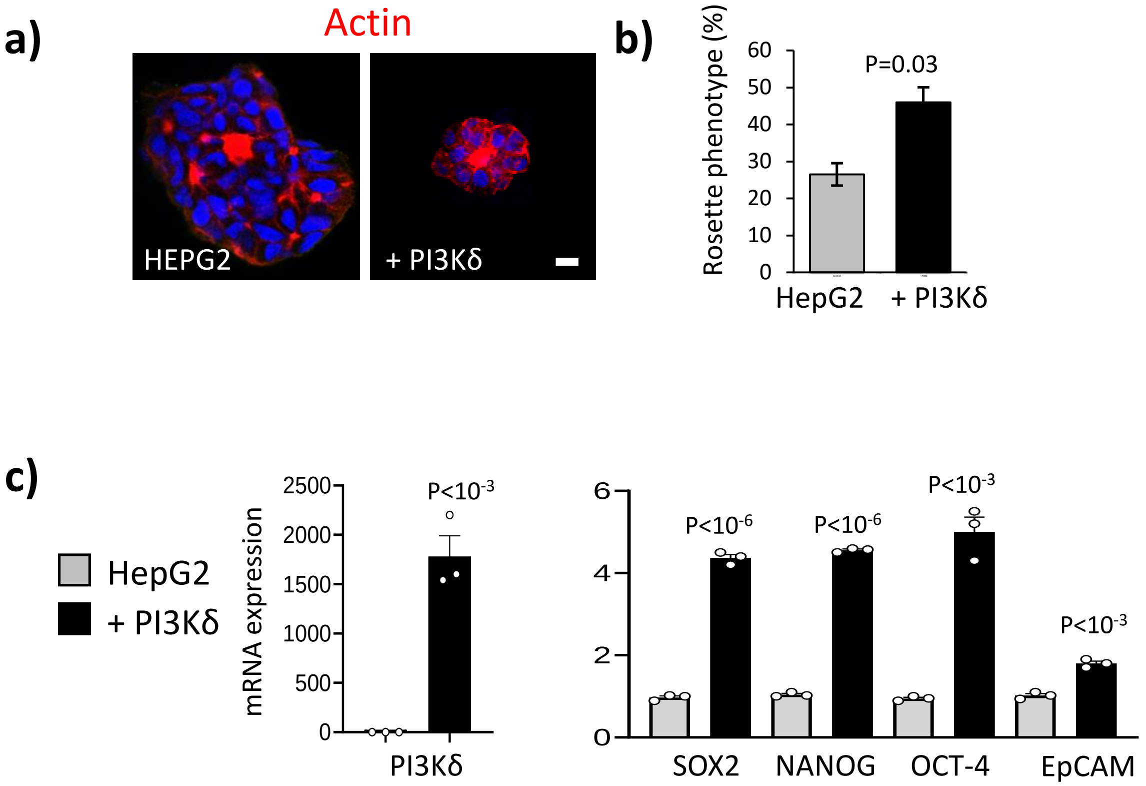
a)
Actin
HEPG2
+ PI3Kδ
b)
Rosette phenotype (%)
P=0.03
HepG2
+ PI3Kδ
c)
HepG2
+ PI3Kδ
mRNA expression
P<10-3
PI3Kδ
P<10-6
P<10-6
P<10-3
P<10-3
SOX2
NANOG
OCT-4
EpCAM

Supplementary Figure 3. PI3Kδ overexpression induces rosette-like structures and stemness factors in HepG2 cells

a) Immunofluorescence staining for the actin microfilaments using phalloidin (red) and nuclei (blue) in HepG2 cells transfected or not with a plasmid coding for PI3Kδ (+PI3Kδ) after 6 days of 3D culture. Scale bar: 10 µm. **b)** Quantification of rosette forming (percentage) between the two conditions after 6 days of 3D culture. **c)** RT-qPCR analysis of different genes expression performed in HepG2 and HepG2+PI3Kδ.(n=3 independent experiments). GADPH was used as housekeeping gene for normalization. All values are expressed as mean±S.E.M.

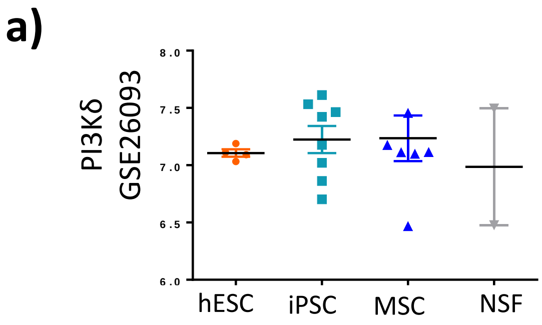
a)
PI3Kδ GSE26093
8.0
7.5
7.0
6.5
6.0
hESC
iPSC
MSC
NSF

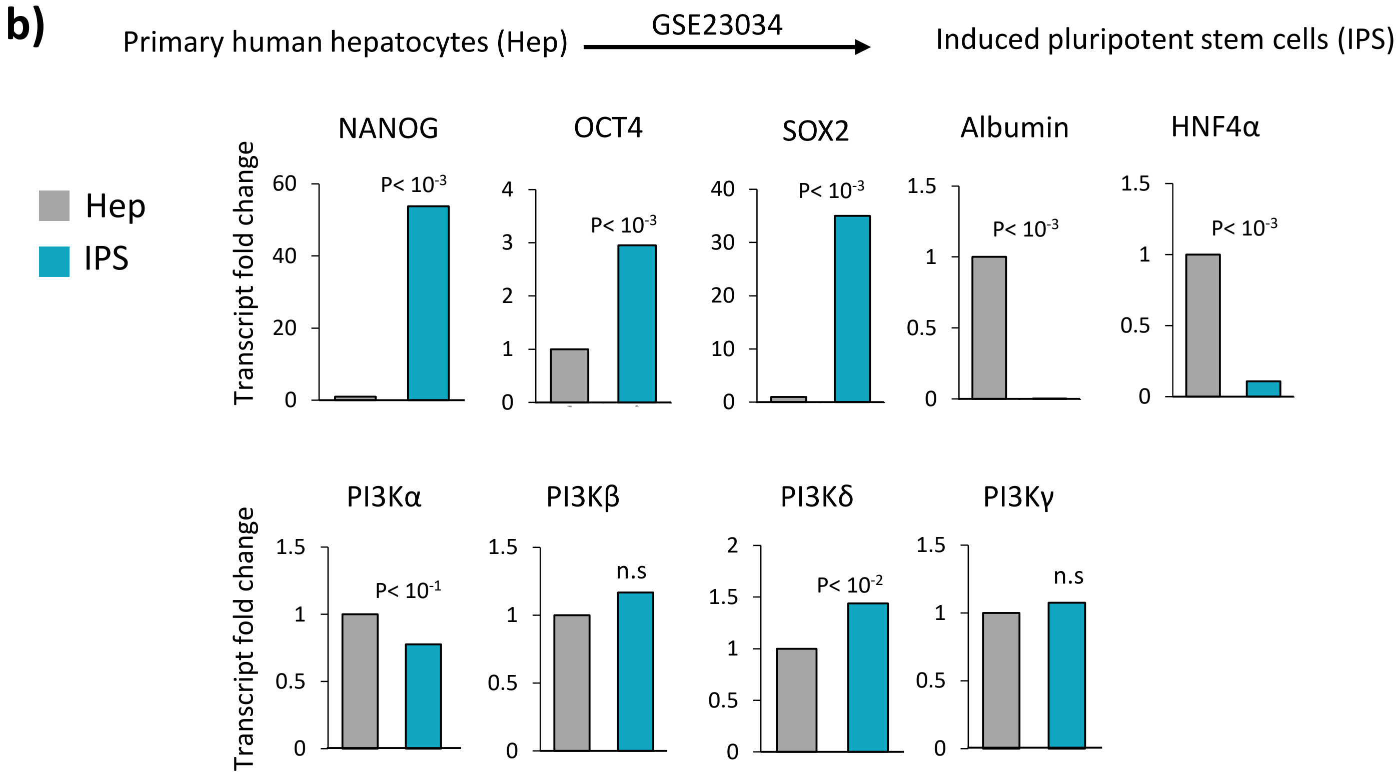
b)
Primary human hepatocytes (Hep)
GSE23034
Induced pluripotent stem cells (IPS)
Hep
IPS
Transcript fold change
NANOG
OCT4
SOX2
Albumin
HNF4α
P< 10-3
PI3Kα
PI3Kβ
PI3Kδ
PI3Kγ
P< 10-1
n.s
P< 10-2

Supplementary Figure 4. Expression profile of PI3Kδ in stem cells

a) Expression of PI3Kδ in human embryonic stem cell (hESC), induced-pluripotent stem cell (iPSC), mesenchymal stem cell (MSC) and fibroblasts using the dataset GSE26093. **b)** Fold change in the expression of the several stem cell, differentiation markers and different class I PI3K isoforms (PI3Kδ, PI3Kα, PI3Kβ, PI3Kγ) in hepatocyte and iPS cells using the dataset GSE23034.

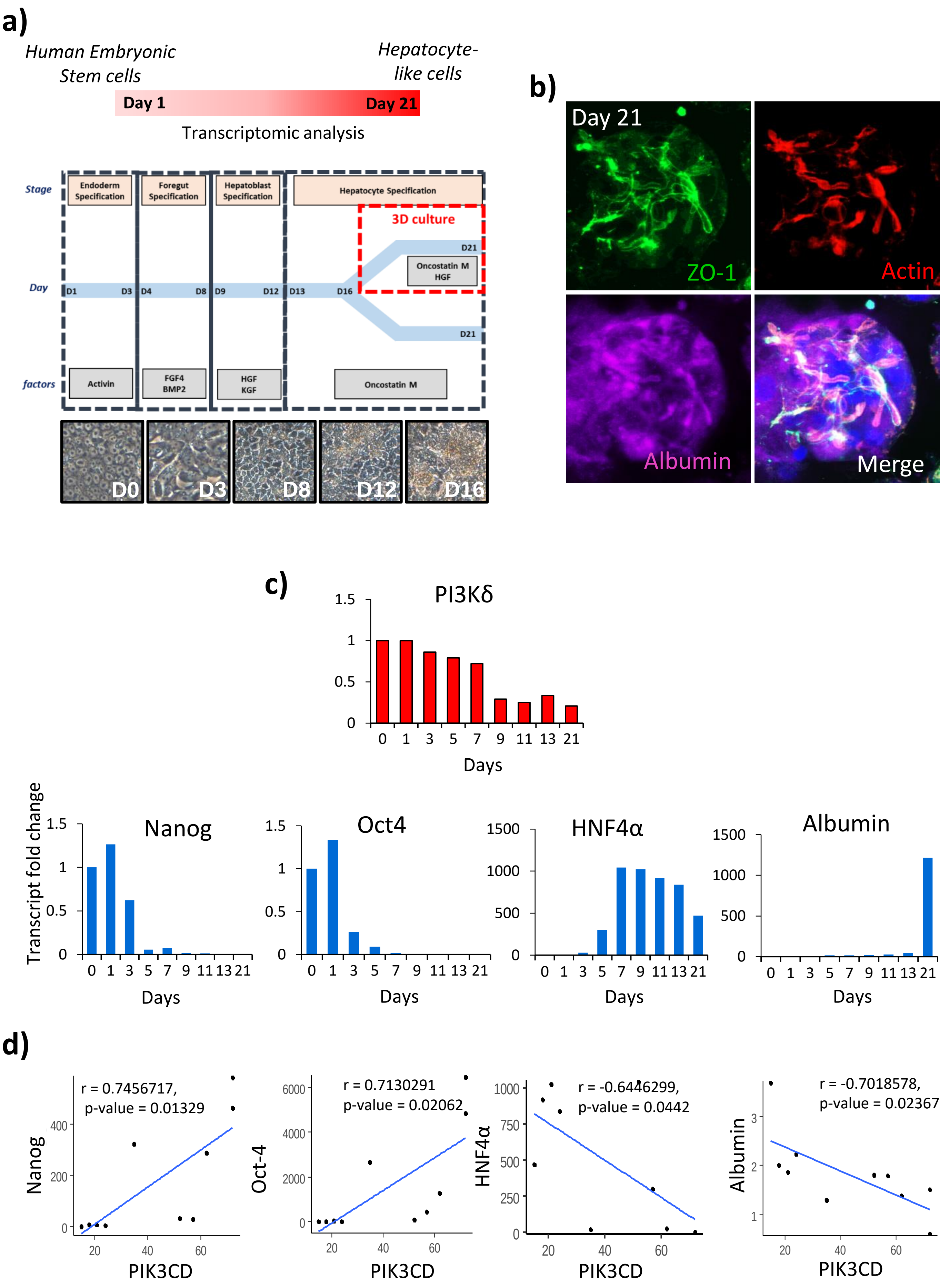
a)
Human Embryonic Stem cells
Hepatocyte-like cells
Day 1
Day 21
Transcriptomic analysis
Stage
Endoderm Specification
Foregut Specification
Hepatoblast Specification
Hepatocyte Specification
3D culture
D21
Oncostatin M HGF
Day
D1 D3 D4 D8 D9 D12 D13 D16
D21
factors
Activin
FGF4 BMP2
HGF KGF
Oncostatin M
D0
D3
D8
D12
D16
b)
Day 21
ZO-1
Actin
Albumin
Merge
c)
PI3Kδ
Days
Transcript fold change
Nanog
Oct4
HNF4α
Albumin
Days
d)
r = 0.7456717, p-value = 0.01329
Nanog
PIK3CD
r = 0.7130291 p-value = 0.02062
Oct-4
PIK3CD
r = -0.6446299, p-value = 0.0442
HNF4α
PIK3CD
r = -0.7018578, p-value = 0.02367
Albumin
PIK3CD

Supplementary Figure 5. Expression profile of PI3Kδ during hESC differentiation

a) Experiment plan for hESC differentiation into hepatocyte-like cells. **b)** 3D reconstruction of an organoid formed after 6 days of 3D culture, from cell plated at day 16 of their differentiation. Organoids were stained for Zonula-occludens 1 (ZO-1, green), actin microfilaments using phalloidin (red), albumin (purple) and nuclei (blue). **c)** Transcription profile of different markers during hESC differentiation into hepatocyte-like cells. **d)** Correlation between expressions of the genes showed in panel (**c**) and PI3Kδ expression.

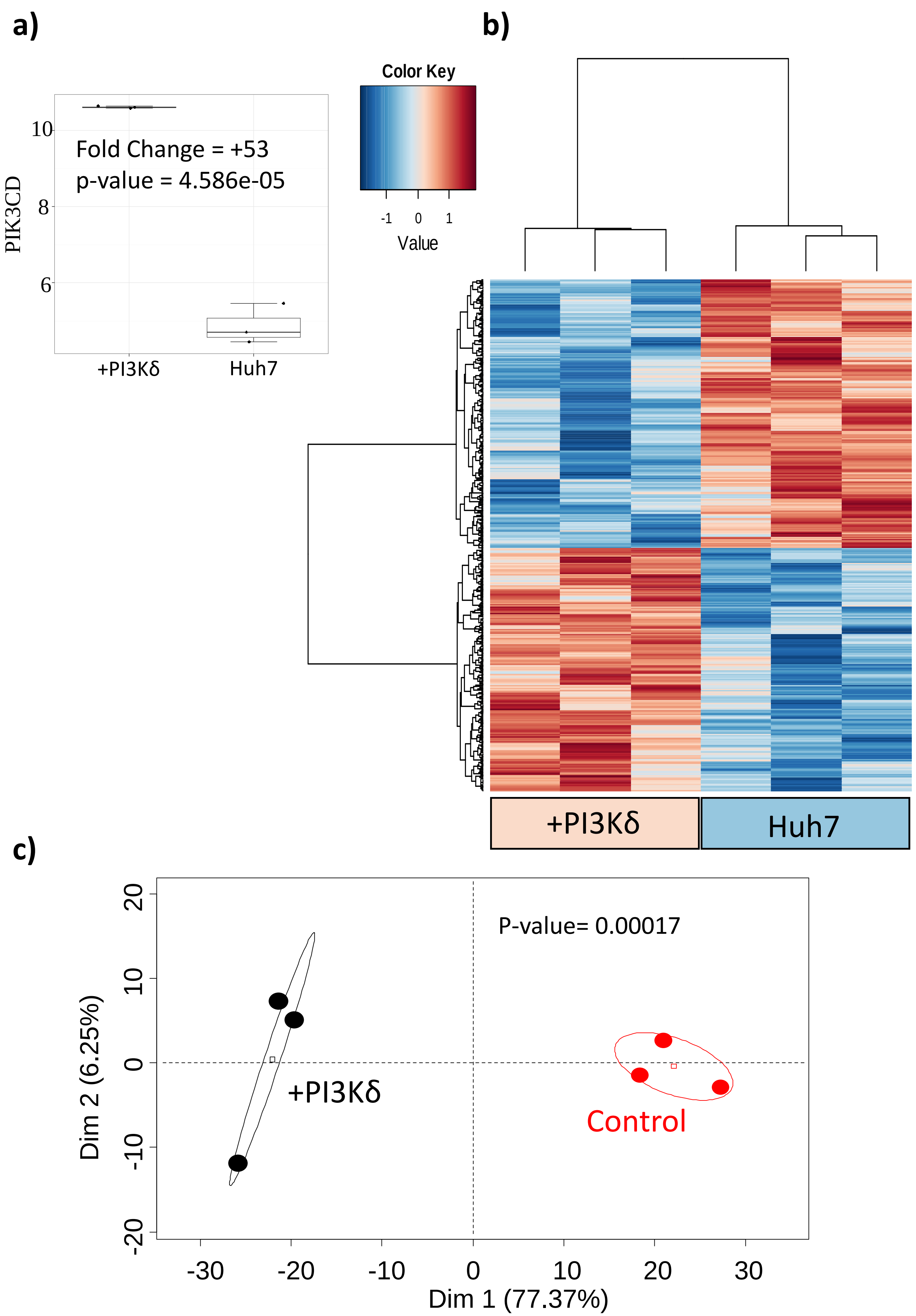
a)
b)
c)
PIK3CD
10
8
6
Fold Change = +53
p-value = 4.586e-05
+PI3Kδ
Huh7
Color Key
-1
0
1
Value
+PI3Kδ
Huh7
P-value= 0.00017
+PI3Kδ
Control
Dim 2 (6.25%)
Dim 1 (77.37%)
20
10
0
-10
-20
-30
-20
-10
0
10
20
30

Supplementary Figure 6. Transcriptomic analysis of Huh7 overexpressing PI3Kδ

a) Boxplot of PI3Kδ expression in Huh7 control and Huh7+PI3Kδ found in the transcriptomics analysis. **b)** Heatmap of the 312 upregulated and 348 down-regulated genes in Huh7+PI3Kδ and unsupervised classification performed on the gene expression profiling between the two conditions**. c)** Unsupervised principal component analysis performed on the gene expression profiling in Huh7 and Huh7+PI3Kδ (the p-value was calculated with group correlation to the first principal axis).

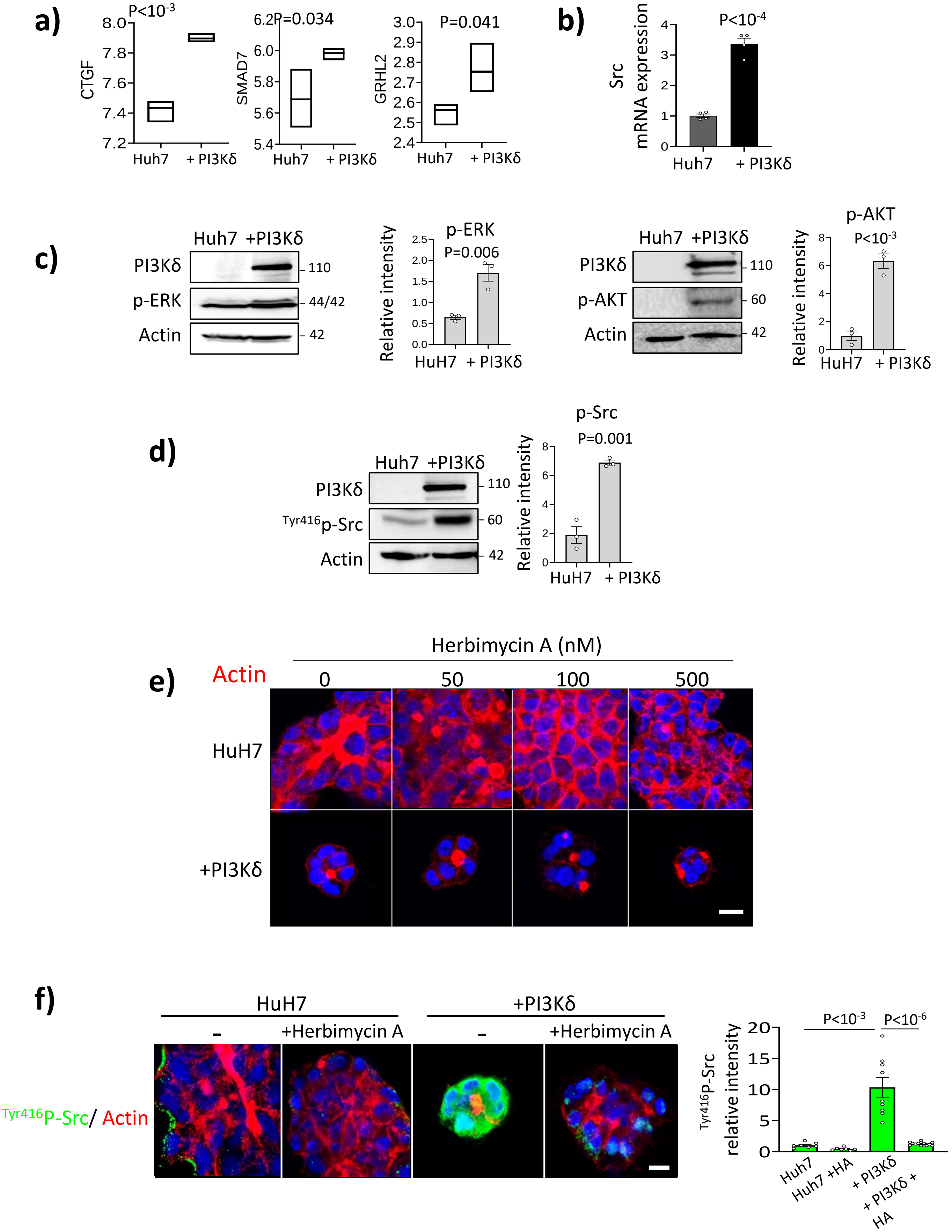
a)
P<10-3
CTGF
8.0
7.8
7.6
7.4
7.2
Huh7
+ PI3Kδ
P=0.034
SMAD7
6.2
6.0
5.8
5.6
5.4
P=0.041
GRHL2
3.0
2.9
2.8
2.7
2.6
2.5
2.4
b)
Src
mRNA expression
P<10-4
c)
Huh7 +PI3Kδ
PI3Kδ
p-ERK
Actin
110
44/42
42
p-ERK
P=0.006
Relative intensity
HuH7
p-AKT
P<10-3
60
d)
p-Src
P=0.001
Tyr416p-Src
e)
Herbimycin A (nM)
Actin
0
50
100
500
HuH7
+PI3Kδ
f)
Tyr416P-Src/ Actin
+Herbimycin A
-
Tyr416p-Src
relative intensity
P<10-3
P<10-6
Huh7
Huh7 +HA
+ PI3Kδ
+ PI3Kδ + HA

Supplementary Figure 7. Effects of Src signaling pathway inhibition on PI3Kδ induced rosette-like structures.

a) Boxplot of CTGF, SMAD7 and GRHL2 expression in the transcriptome from a Huh7 and Huh7+PI3Kδ (the p-value was calculated using an unpaired two-sided Student's t test) based on 10 p-values. **b)** RT-qPCR analysis of SRC expression performed in Huh7 and Huh7+PI3Kδ organoids after 6 days of 3D culture in two independent experiments performed in duplicate. RPLP0 was used as the housekeeping gene for normalization. **c)** Immunoblot analysis of p-ERK and p-AKT in Huh7 and Huh7+PI3Kδ with the quantification of the relative intensity (n=3 experiments). **d)** Immunoblot analysis of p-Src in Huh7 and Huh7+PI3Kδ with the quantification of the relative intensity (n=3 experiments). **e)** Immunofluorescence staining for actin microfilaments using phalloidin (red) and nuclei (blue) in Huh7 control and Huh7+PI3Kδ organoids after 6 days of 3D culture treated or not with the Src inhibitor (Herbimycin A) at different concentrations (0-500 nM) as indicated in the panel. Scale bar: 10 µm. **f)** Immunofluorescence staining after 6 days of 3D culture for p-SRC (green), actin microfilaments using phalloidin (red) and nuclei (blue) in Huh7 control and Huh7+PI3Kδ organoids treated or not with herbimycin A (Src inhibitor) at 100 nM. Quantification of its relative intensity (right). Each dot of the graph corresponds to an organoid. Scale bar: 10µm.

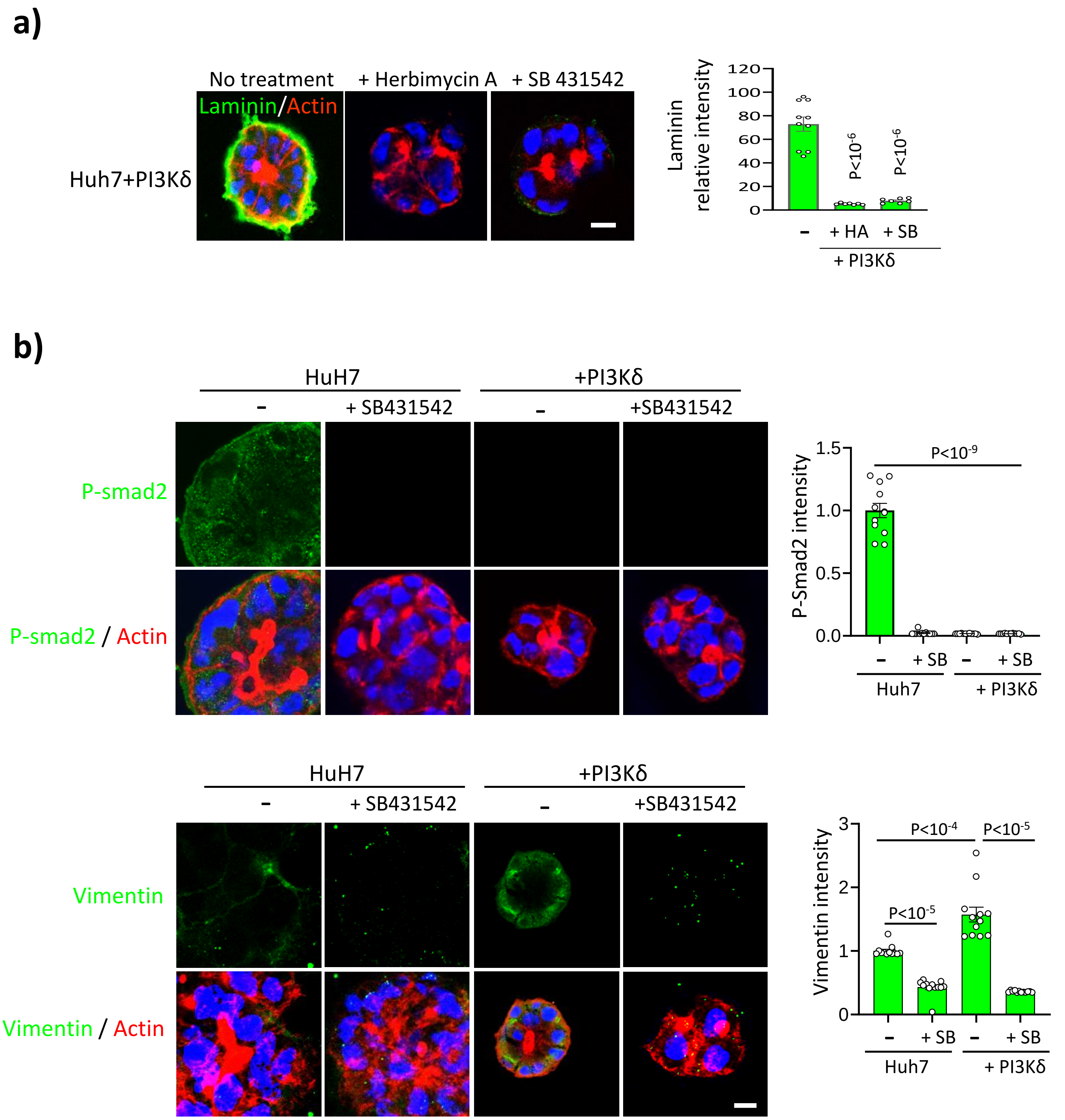
a)
No treatment
+ Herbimycin A
+ SB 431542
Laminin/Actin
Huh7+PI3Kδ
Laminin relative intensity
120
100
80
60
40
20
0
P<10-6
P<10-6
–
+ HA
+ SB
+ PI3Kδ
b)
HuH7
+PI3Kδ
-
+ SB431542
-
+SB431542
P-smad2
P-smad2 / Actin
P-Smad2 intensity
1.5
1.0
0.5
0.0
P<10-9
–
+ SB
–
+ SB
Huh7
+ PI3Kδ
HuH7
+PI3Kδ
-
+ SB431542
-
+SB431542
Vimentin
Vimentin / Actin
Vimentin intensity
3
2
1
0
P<10-4
P<10-5
P<10-5
–
+ SB
–
+ SB
Huh7
+ PI3Kδ

Supplementary Figure 8. Effects of TGFβ signaling pathway inhibition on PI3Kδ induced rosette-like structures.

a) Immunofluorescence staining for laminin (green), actin microfilaments using phalloidin (red) and nuclei (blue) of Huh7+PI3Kδ organoids treated with 100 nM of Herbimycin A or 2 µM of SB431542 after 6 days of 3D culture. Scale bar: 10 µm. Quantification of the relative intensity (right). Each dot of the graph corresponds to an organoid. All values are expressed as mean±S.E.M. **b)** Immunofluorescence staining after 6 days of 3D culture for p-Smad2 or Vimentin (green), actin microfilaments using phalloidin (red) and nuclei (blue) in Huh7 control and Huh7+PI3Kδ organoids treated or not with SB431542 (TGFβ inhibitor) at 2 µM. Scale bar: 10µm. Quantification of the relative intensity (right). Each dot of the graph corresponds to an organoid. All values are expressed as mean±S.E.M.

a)

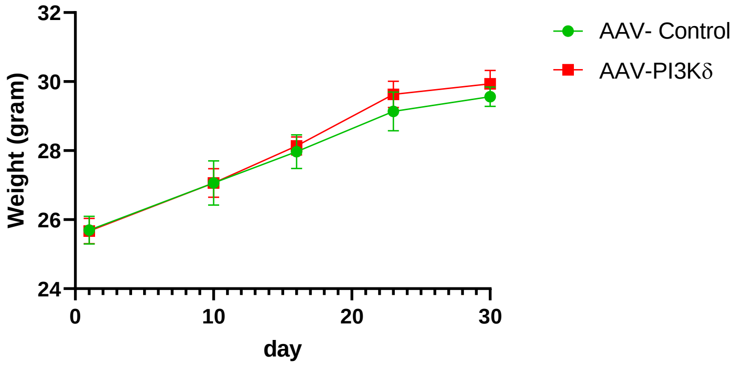
32
30
28
26
24
Weight (gram)
0
10
20
30
day
AAV- Control
AAV-PI3Kδ

b)

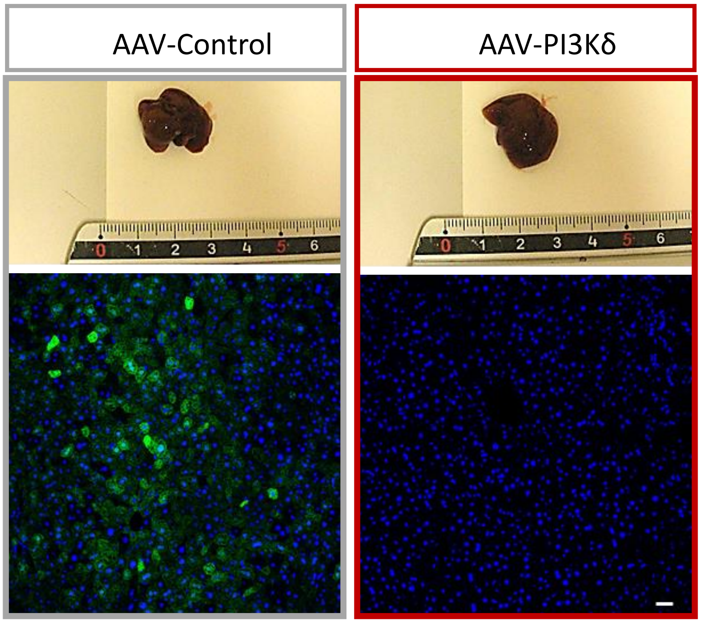
AAV-Control
AAV-PI3Kδ

Supplementary Figure 9. Effect of PI3Kδ overexpression in mouse liver

a) Analysis of the weight gain between AAV-control and AAV- PI3Kδ mice (n= 4 mice per condition). **b)** (Top) Liver of mice infected AAV-Control and AAV-PI3Kδ. (Bottom) immunofluorescence staining of EGFP in the two conditions.

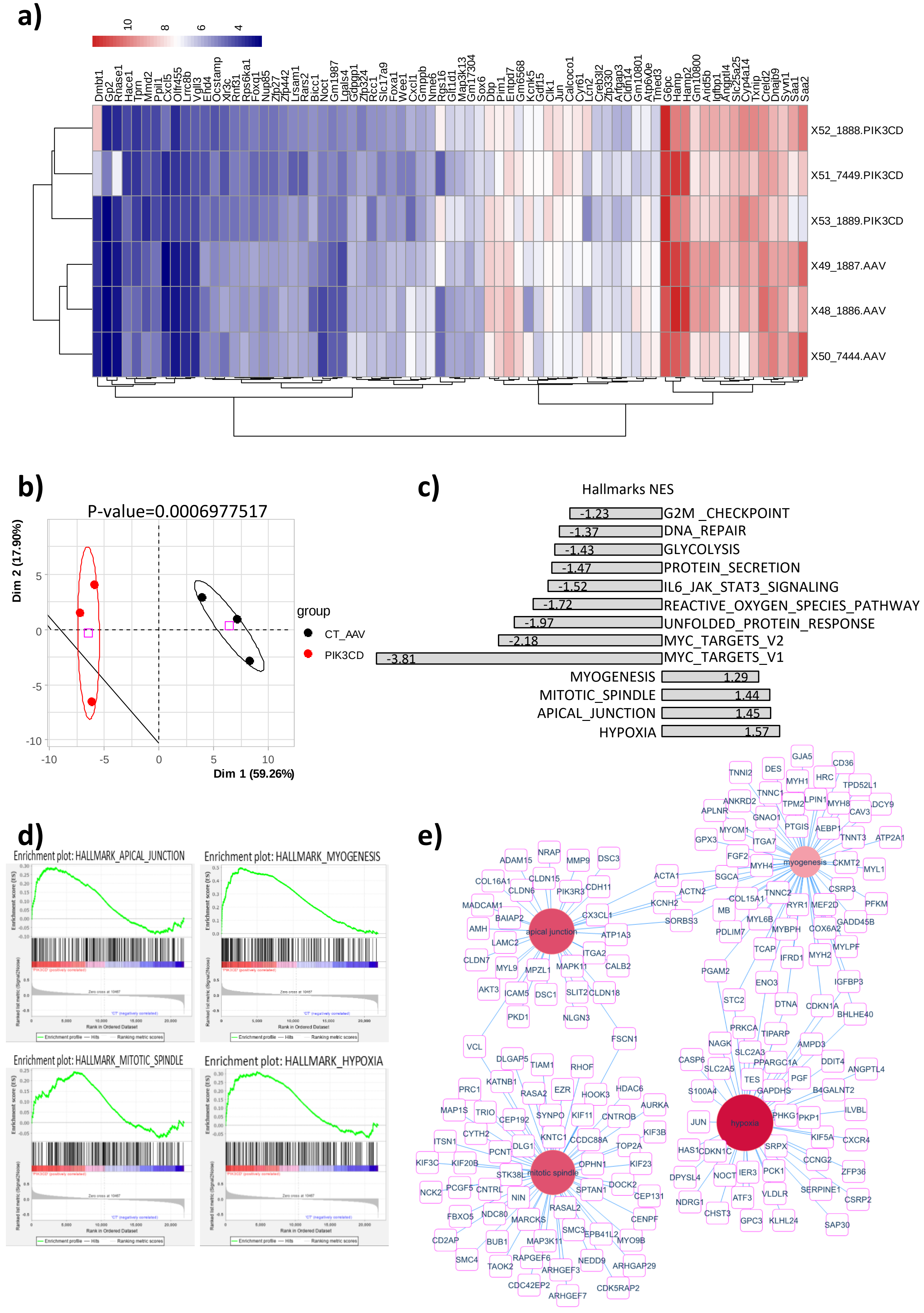

a)
10
8
6
4
Dmbt1 Gp2 Rnase1 Hace1 Tprn Mmd2 Ppil1 Cxcl5 Olfr455 Lrrc8b Vgll3 Efhd4 Ocstamp Xlr3c Rnf31 Rps6ka1 Foxq1 Nup85 Zfp27 Zfp442 Lrsam1 Rars2 Bicc1 Noct Gm1987 Lgals4 Gdpgp1 Zfp324 Rcc1 Slc17a9 Foxa1 Wee1 Cxcl1 Gmppb Nme6 Rgs16 Gtf1d1 Map3k13 Gm17304 Sox6 Dbp Pim1 Entpd7 Gm6568 Kcnk5 Gdf15 Clk1 Jun Calcoco1 Cyr61 Lcn2 Creb3l2 Zfp330 Arfgap3 Cldn14 Gm10801 Atp6v0e Tmed3 G6pc Hamp Hamp2 Gm10800 Arid5b Igfbp1 Angptl4 Slc25a25 Cyp4a14 Txnip Creld2 Dnajb9 Syvn1 Saa1 Saa2
X52_1888.PIK3CD
X51_7449.PIK3CD
X53_1889.PIK3CD
X49_1887.AAV
X48_1886.AAV
X50_7444.AAV
b)
P-value=0.0006977517
Dim 2 (17.90%)
Dim 1 (59.26%)
group
CT_AAV
PIK3CD
c)
Hallmarks NES
-1.23 G2M _CHECKPOINT
-1.37 DNA_REPAIR
-1.43 GLYCOLYSIS
-1.47 PROTEIN_SECRETION
-1.52 IL6_JAK_STAT3_SIGNALING
-1.72 REACTIVE_OXYGEN_SPECIES_PATHWAY
-1.97 UNFOLDED_PROTEIN_RESPONSE
-2.18 MYC_TARGETS_V2
-3.81 MYC_TARGETS_V1
MYOGENESIS 1.29
MITOTIC_SPINDLE 1.44
APICAL_JUNCTION 1.45
HYPOXIA 1.57
d)
Enrichment plot: HALLMARK_APICAL_JUNCTION
Enrichment plot: HALLMARK_MYOGENESIS
Enrichment plot: HALLMARK_MITOTIC_SPINDLE
Enrichment plot: HALLMARK_HYPOXIA
Enrichment score (ES)
Ranked list metric (Signal2Noise)
Rank in Ordered Dataset
Enrichment profile — Hits — Ranking metric scores
e)
apical junction
myogenesis
mitotic spindle
hypoxia

Supplementary Figure 10. PI3Kδ tumor transfection impact on distinct cellular functions

a) Heatmap of the genes regulated by PI3Kδ and classification by Euclidean distances. **b)** Principal component analysis on PI3Kδ regulated genes (p-value by Pearson correlation of group stratification). **c)** Barplot of gene set enrichment performed on hallmarks database from Broad Institute **d)** Hallmarks gene set up regulated in PI3Kδ condition. **e)** Functional enriched network performed on Hallmarks cellular function up regulation in PI3Kδ condition.

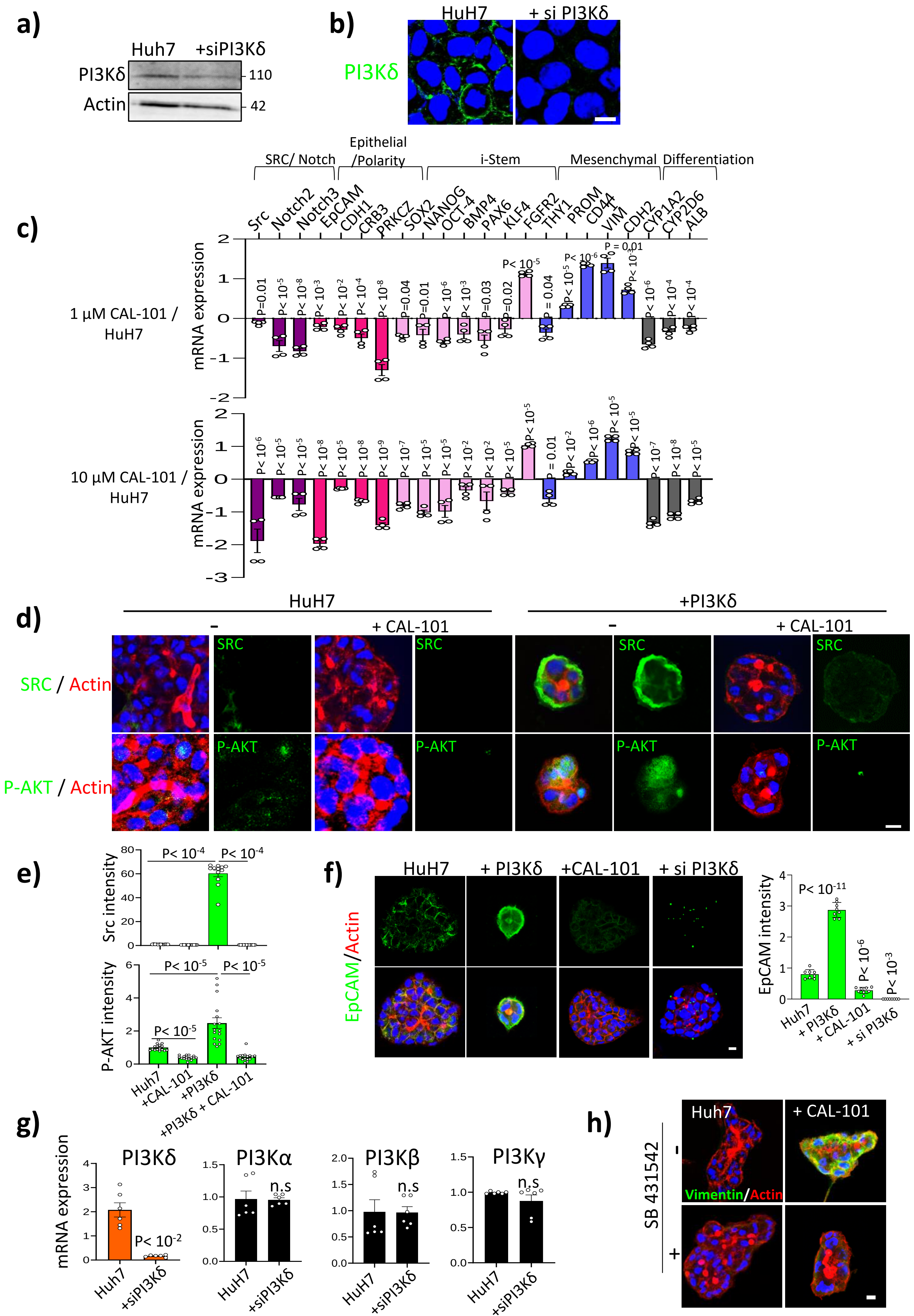
a)
Huh7
+siPI3Kδ
PI3Kδ
Actin
110
42
b)
HuH7
+ si PI3Kδ
PI3Kδ
c)
SRC/ Notch
Epithelial /Polarity
i-Stem
Mesenchymal
Differentiation
Src Notch2 Notch3 EpCAM CDH1 CRB3 PRKCZ SOX2 NANOG OCT-4 BMP4 PAX6 KLF4 FGFR2 THY1 PROM CD44 VIM CDH2 CYP1A2 CYP2D6 ALB
1 µM CAL-101 / HuH7
10 µM CAL-101 / HuH7
mRNA expression
d)
HuH7
+PI3Kδ
+ CAL-101
SRC / Actin
P-AKT / Actin
SRC
P-AKT
e)
Src intensity
P-AKT intensity
Huh7
+CAL-101
+PI3Kδ
+PI3Kδ + CAL-101
f)
HuH7
+ PI3Kδ
+CAL-101
+ si PI3Kδ
EpCAM/Actin
EpCAM intensity
Huh7
g)
PI3Kδ
PI3Kα
PI3Kβ
PI3Kγ
mRNA expression
Huh7
+siPI3Kδ
n.s
h)
Huh7
+ CAL-101
SB 431542
Vimentin/Actin

Supplementary Figure 11: Effects of PI3Kδ inhibition in Huh7 cells morphology and EMT markers

a) Immunoblot analysis of PI3Kδ in Huh7 control and Huh7+siPI3Kδ. **b)** Immunofluorescence staining for PI3Kδ (green) in 2D culture of Huh7 control and Huh7+siPI3Kδ. Scale bar: 10µm. **c)** RT-qPCR analysis of different genes expression performed in organoids of Huh7 control and Huh7+CAL-101 (1-10 µM) after 6 days of 3D culture in two independent experiments performed in duplicate. Data are presented as $\log_{10}$ mRNA fold change in the different conditions compared to Huh7 control. RPLP0 was used as the housekeeping gene for normalization. **d)** Immunofluorescence staining after 6 days of 3D culture for Src and p-AKT (green) , actin microfilaments using phalloidin (red) and nuclei (blue) in Huh7 control and Huh7+PI3Kδ organoids treated or not with CAL-101 at 5 µM. Scale bar: 10µm. **e)** Quantification of relative intensity of Src and p-AKT. Each dot of the graph corresponds to an organoid. **f)** Immunofluorescence staining for EpCAM (green), actin microfilaments using phalloidin (red) and nuclei (blue) as indicated in the panel in organoids of Huh7 control, Huh7+PI3Kδ, Huh7+CAL-101 (5µM) and Huh7+siPI3Kδ and quantification of relative intensity of EpCAM. Scale bar: 10µm. Each dot of the graph corresponds to an organoid. **g)** RT-qPCR analysis of the different class I PI3Ks isoforms expression performed in HuH7 control and Huh7+siPI3Kδ in three independent experiments performed in duplicate. RPLP0 was used as the housekeeping gene for normalization. **h)** Immunofluorescence staining for vimentin (green), actin microfilaments using phalloidin (red) and nuclei (blue) in organoids of Huh7 and Huh7+CAL-101 (5µM) treated or not with 2 µM of SB431542 during 6 days of 3D culture. Scale bar: 10 µm. All values are expressed as mean±S.E.M.

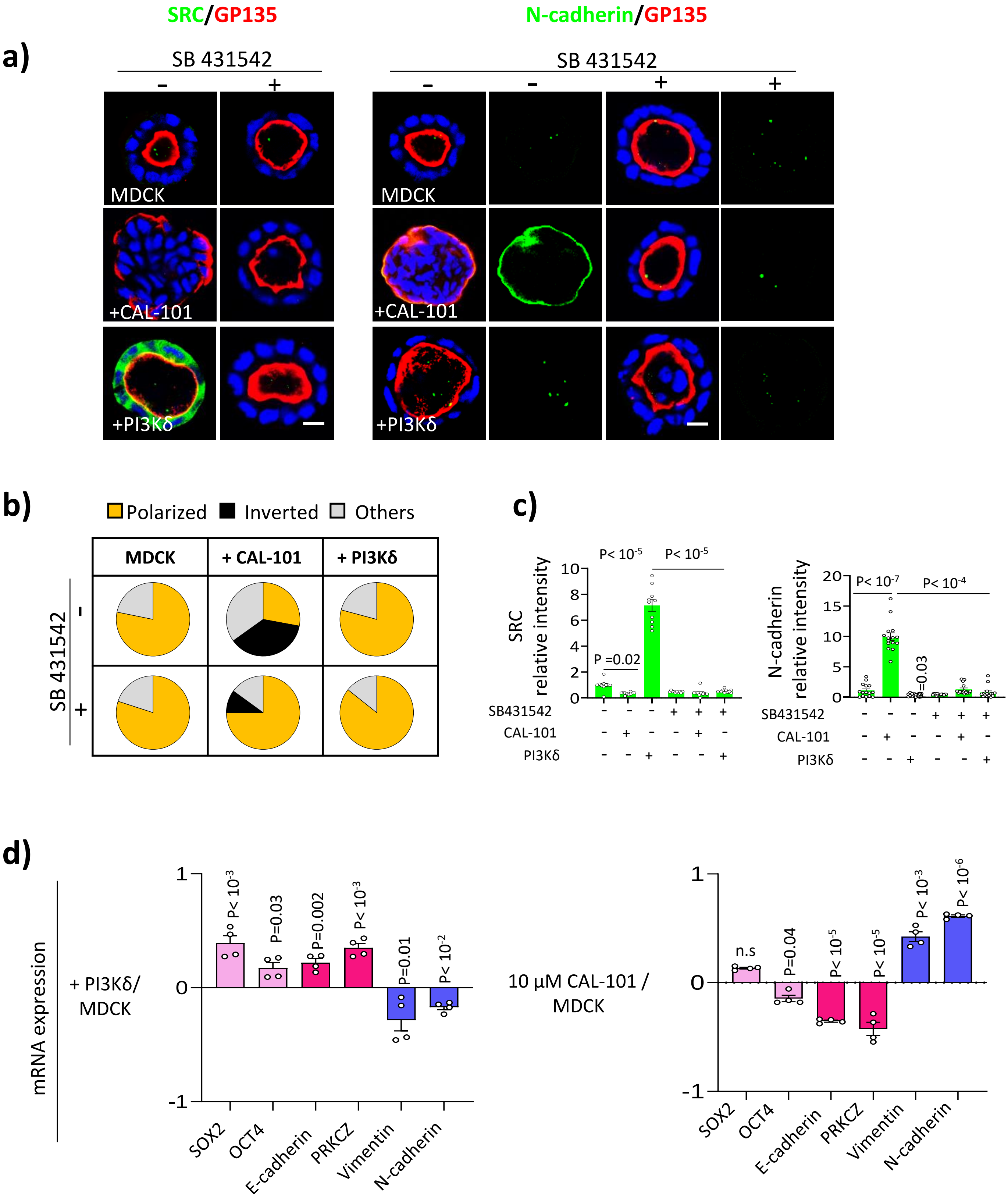

a)
SRC/GP135
N-cadherin/GP135
SB 431542
- +
SB 431542
- - + +
MDCK
+CAL-101
+PI3Kδ
MDCK
+CAL-101
+PI3Kδ
b)
Polarized Inverted Others
MDCK
+ CAL-101
+ PI3Kδ
SB 431542
-
+
c)
SRC relative intensity
P< 10-5
P< 10-5
P =0.02
SB431542
CAL-101
PI3Kδ
N-cadherin relative intensity
P< 10-7
P< 10-4
P=0.03
SB431542
CAL-101
PI3Kδ
d)
mRNA expression
+ PI3Kδ/ MDCK
P< 10-3
P=0.03
P=0.002
P< 10-3
P=0.01
P< 10-2
SOX2
OCT4
E-cadherin
PRKCZ
Vimentin
N-cadherin
10 µM CAL-101 / MDCK
n.s
P=0.04
P< 10-5
P< 10-5
P< 10-3
P< 10-6
SOX2
OCT4
E-cadherin
PRKCZ
Vimentin
N-cadherin

Supplementary Figure 12. Effects of PI3Kδ activity modulation and TGFβ signaling pathway inhibition in MDCK organoids

a) Immunofluorescence staining for Src and N-cadherin (green), GP135 (red) and nuclei (blue) in MDCK control, MDCK+PI3Kδ and MDCK+CAL-101 (10 µM) organoids after 6 days of 3D culture and treated with 2 µM of SB431542. Scale bar: 10µm. **b)** Quantification of the phenotypes percentage in the different conditions. **c)** Quantification of Src and N-cadherin relative intensity in the different conditions presented in (**a**) each dot of the graph corresponds to an organoid. **d)** RT-qPCR analysis of different genes expression performed in MDCK control, MDCK+PI3Kδ and MDCK+CAL-101 (10 µM) organoids after 6 days of 3D culture in two independent experiments performed in duplicate. Data are presented as $\log_{10}$ mRNA fold change in the different conditions compared to MDCK. Canis-GAPDH was used as the housekeeping gene for normalization. All values are expressed as mean±S.E.M.

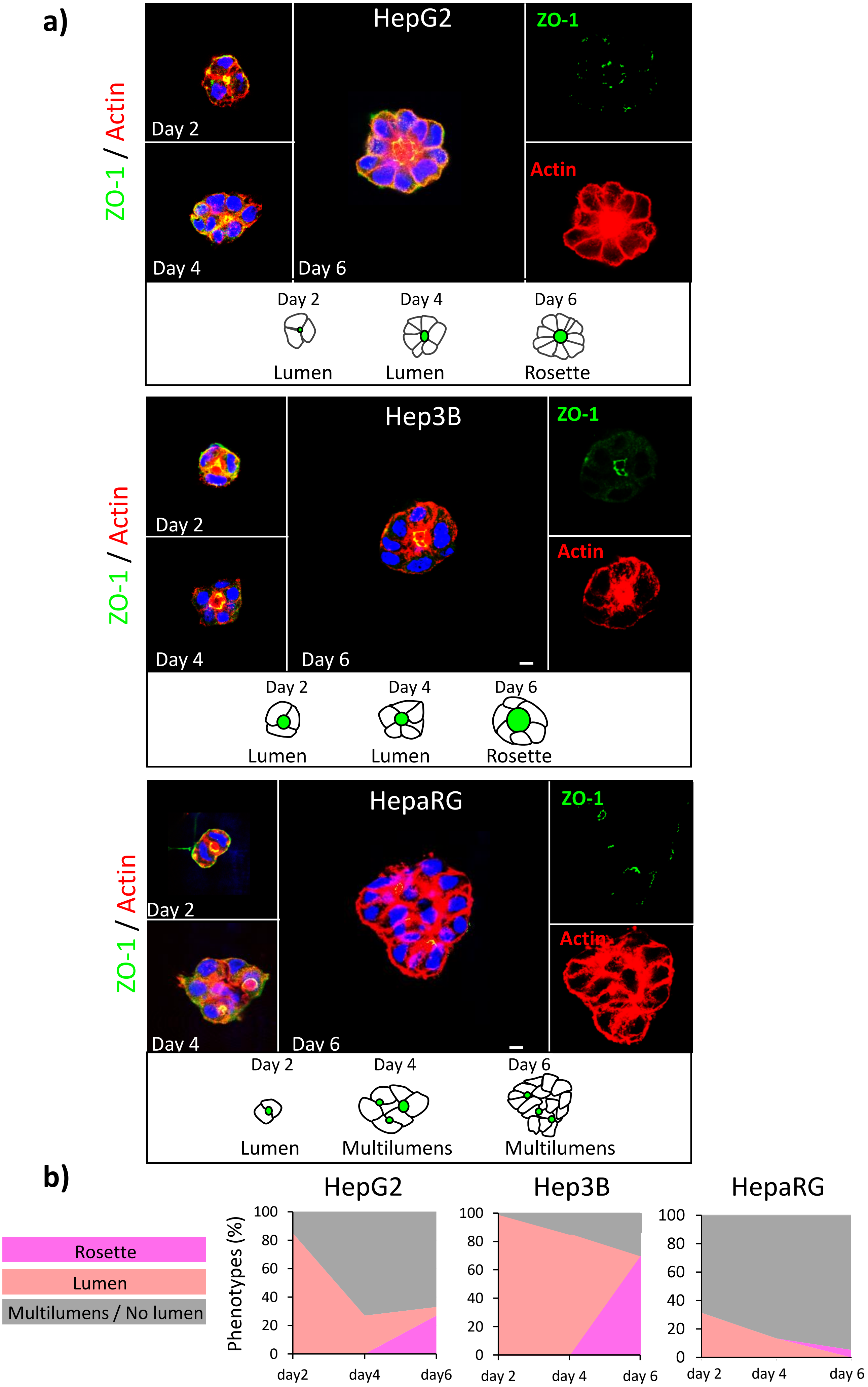

a)
HepG2
ZO-1 / Actin
ZO-1
Actin
Day 2
Day 4
Day 6
Lumen
Lumen
Rosette
Hep3B
Lumen
Lumen
Rosette
HepaRG
Lumen
Multilumens
Multilumens
b)
Rosette
Lumen
Multilumens / No lumen
Phenotypes (%)
HepG2
Hep3B
HepaRG
0
20
40
60
80
100
day2
day4
day6
day 2
day 4
day 6

Supplementary Figure 13. Lumen formation kinetics in different hepatic cell lines

a) Time-course analysis of lumen formation in HepG2, Hep3B and HepaRG plated in 3D Matrigel-matrix and stained after 2, 4 or 6 days for Zonula-occludens 1 (ZO-1, green), actin microfilaments using phalloidin (red) and nuclei using Hoechst (blue). Scale bar: 10 μm. **b)** Quantification of the phenotypes percentage over the days of 3D culture. All values are expressed as mean±S.E.M.

a)

Differentiation markers

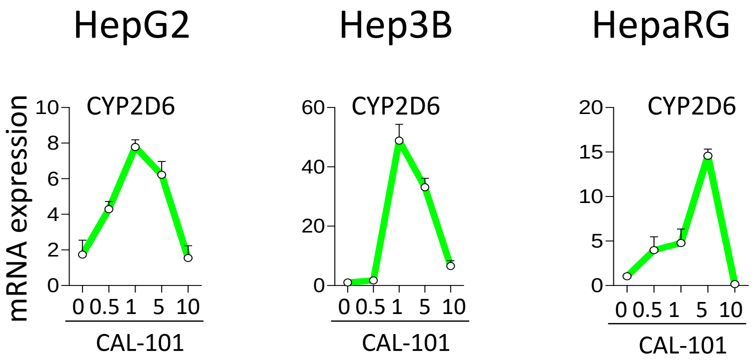

b)

HepG2	0.5	1	5	10
Albumin	P=0.01	$P<10^{-7}$	$P<10^{-7}$	n.s
CYP1A2	$P<10^{-2}$	$P<10^{-5}$	$P<10^{-6}$	n.s
SOX2	$P<10^{-6}$	$P<10^{-6}$	$P<10^{-6}$	$P<10^{-6}$
Nanog	$P<10^{-4}$	$P<10^{-3}$	$P<10^{-3}$	$P<10^{-3}$
CD44	n.s	n.s	n.s	P=0.01
N-cadherin	n.s	n.s	n.s	$P<10^{-3}$
smad7	P=0.03	$P<10^{-6}$	P=0.02	$P<10^{-7}$
Smad3	n.s	n.s	P=0.04	$P<10^{-3}$
CYP2D6	P=0.02	$P<10^{-3}$	$P<10^{-2}$	n.s

Hep3B	0.5	1	5	10
Albumin	P=0.03	$P<10^{-5}$	$P<10^{-3}$	n.s
CYP1A2	n.s	$P<10^{-6}$	$P<10^{-5}$	n.s
SOX2	$P<10^{-2}$	$P<10^{-2}$	$P<10^{-6}$	P=0.03
Nanog	$P<10^{-3}$	$P<10^{-2}$	$P<10^{-3}$	$P<10^{-3}$
CD44	n.s	P=0.02	P=0.01	$P<10^{-3}$
N-cadherin	n.s	n.s	n.s	$P<10^{-3}$
smad7	$P<10^{-3}$	$P<10^{-3}$	$P<10^{-3}$	$P<10^{37}$
Smad3	$P<10^{-3}$	$P<10^{-2}$	P=0.04	P=0.03
CYP2D6	P=0.05	$P<10^{-3}$	$P<10^{-3}$	P=0.05

HepaRG	0.5	1	5	10
Albumin	$P<10^{-6}$	P=0.004	$P<10^{-6}$	$P<10^{-5}$
CYP1A2	P=0.05	$P<10^{-3}$	$P<10^{-6}$	P=0.03
SOX2	P=0.01	$P<10^{-3}$	$P<10^{-3}$	$P<10^{-6}$
Nanog	$P<10^{-5}$	$P<10^{-7}$	$P<10^{-7}$	P=0.03
CD44	n.s	n.s	n.s	$P<10^{-3}$
N-cadherin	n.s	n.s	n.s	$P<10^{-3}$
smad7	$P<10^{-3}$	$P<10^{-3}$	P=0.04	P=0.03
Smad3	n.s	n.s	n.s	P=0.02
CYP2D6	n.s	P=0.04	$P<10^{-7}$	$P<10^{-3}$

Supplementary Figure 14. The inhibition of PI3Kδ activity increases hepatocyte differentiation genes in different cell lines.

a) RT-qPCR analysis of CYP2D6 expression in HepG2, Hep3B and HepaRG cells plated in 3D culture and treated with different doses of CAL-101 after 6 days in two independent experiments performed in duplicate; RPLP0 was used as the housekeeping gene for normalization. b**)** Table representing the p-values of the different genes expression between the organoids treated at different doses of CAL-101 and untreated organoids in the different used cell lines measured by RT-qPCR corresponding to graph presented in figure 6 (panel e) and the panel (**a)** of the supplementary figure 13. All values are expressed as mean±S.E.M.

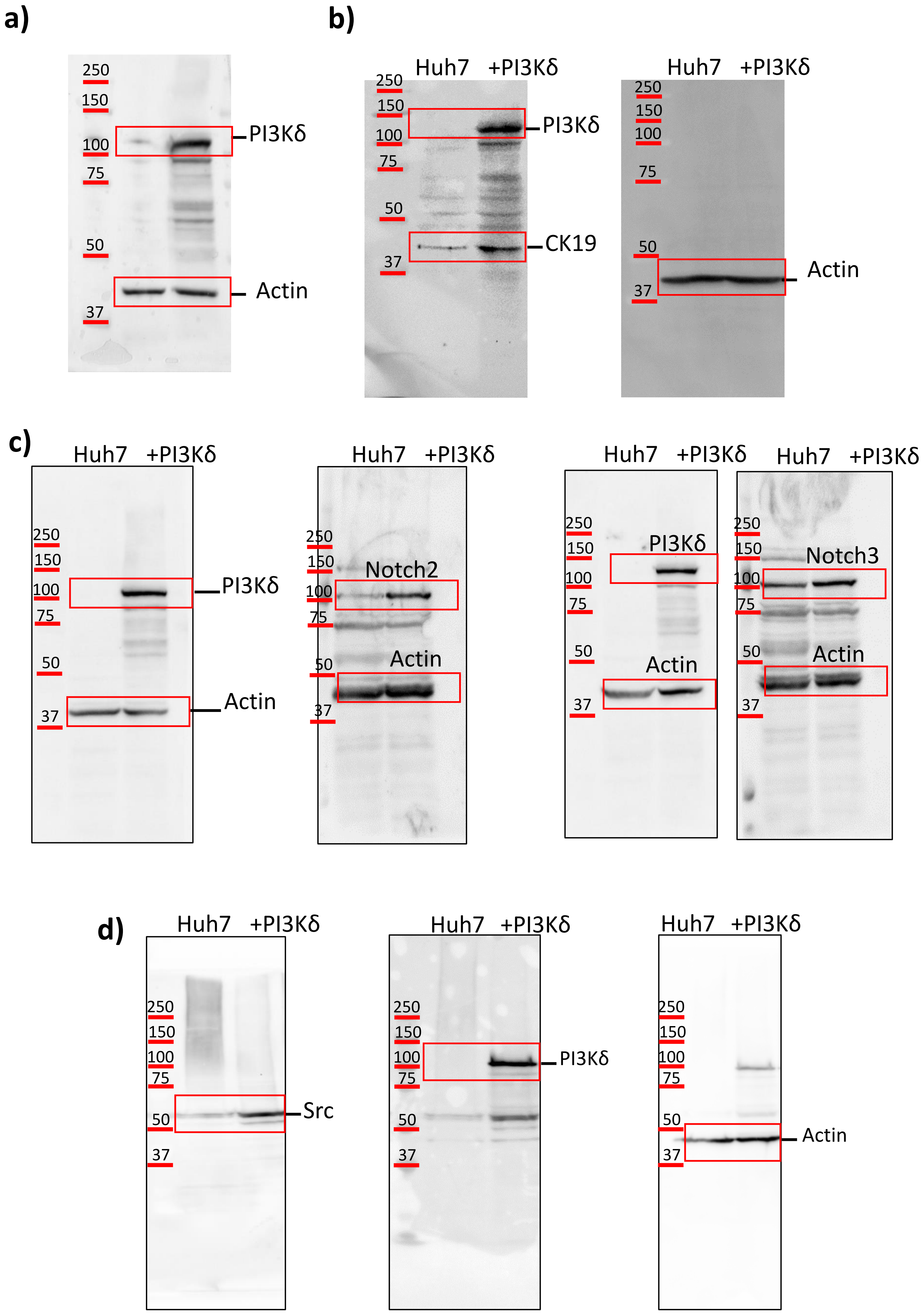
a)
250
150
100
75
50
37
PI3Kδ
Actin
b)
Huh7 +PI3Kδ
PI3Kδ
CK19
Actin
c)
Huh7 +PI3Kδ
PI3Kδ
Notch2
Notch3
Actin
d)
Src
PI3Kδ
Actin

Supplementary Figure 15. Original full blots for the cropped images shown in Supplementary Fig.1a and Fig.2d, Fig,3f **a)** PI3Kδ and β-actin **b)** PI3Kδ, CK19 and β-actin **c)** PI3Kδ, Notch2, β-actin and PI3Kδ, Notch3 and β-actin. **d)** Src, PI3Kδ and β-actin

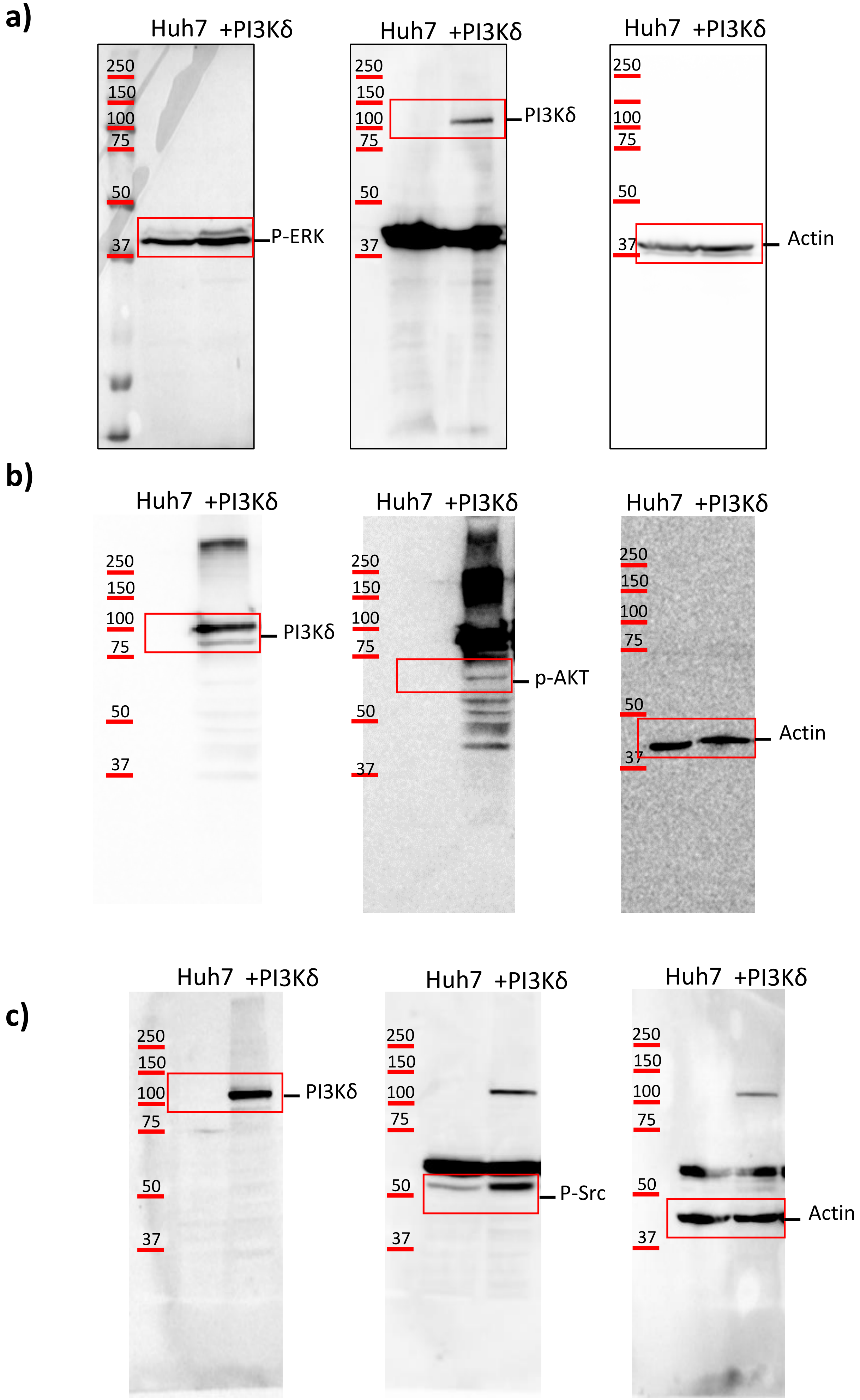
a)
Huh7 +PI3Kδ
250
150
100
75
50
37
P-ERK
PI3Kδ
Actin
b)
Huh7 +PI3Kδ
PI3Kδ
p-AKT
Actin
c)
Huh7 +PI3Kδ
PI3Kδ
P-Src
Actin

Supplementary Figure 16. Original full blots for the cropped images shown in Supplementary Fig.7c and e **a)** p-ERK, PI3Kδ and β-actin. **b)** p-AKT, PI3Kδ and β-actin **c)** p-Src, PI3Kδ and β-actin

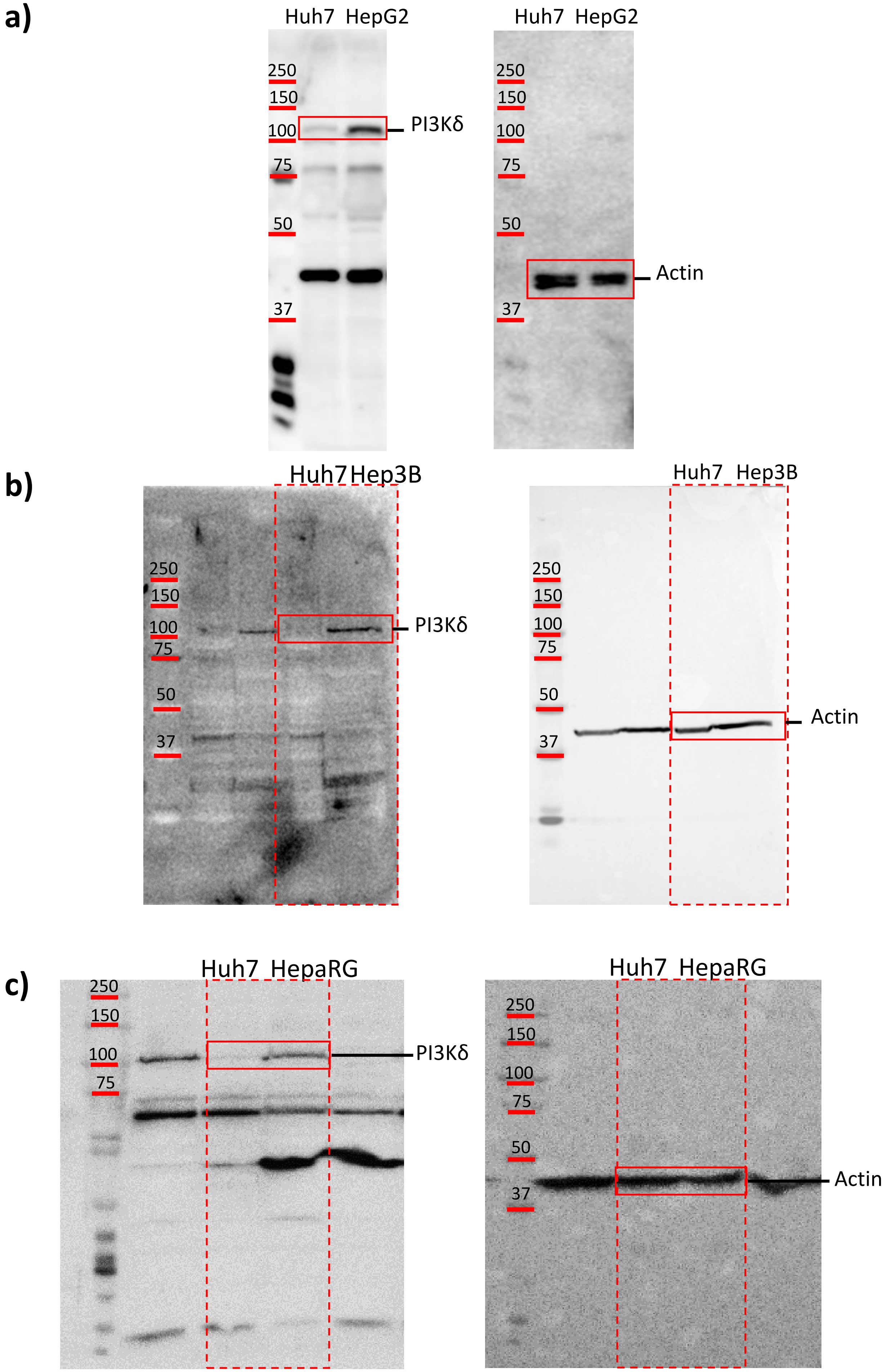

a)
Huh7 HepG2
250
150
100
75
50
37
PI3Kδ
Huh7 HepG2
250
150
100
75
50
37
Actin
b)
Huh7 Hep3B
250
150
100
75
50
37
PI3Kδ
Huh7 Hep3B
250
150
100
75
50
37
Actin
c)
Huh7 HepaRG
250
150
100
75
PI3Kδ
Huh7 HepaRG
250
150
100
75
50
37
Actin

Supplementary Figure 17. Original full blots for the cropped images shown in Fig.6a **a)** PI3Kδ and β-actin **b)** PI3Kδ and β-actin **c)** PI3Kδ and β-actin.

a)

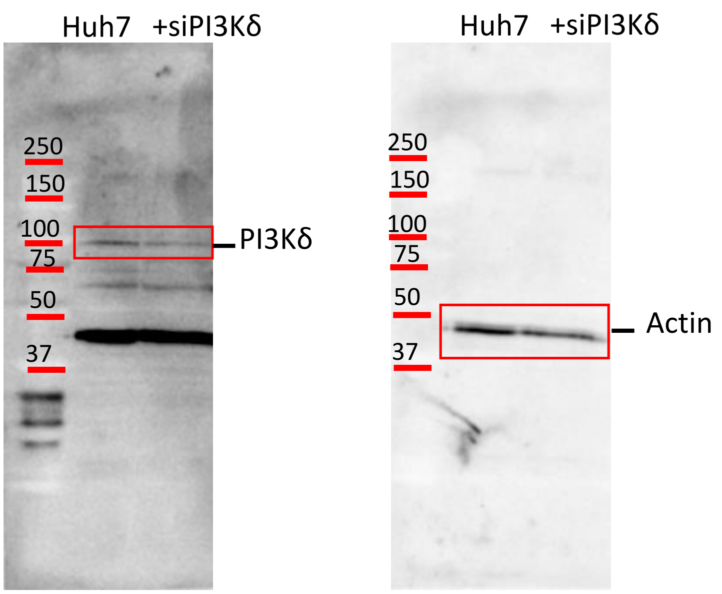
Huh7 +siPI3Kδ
250
150
100
75
50
37
PI3Kδ
Huh7 +siPI3Kδ
250
150
100
75
50
37
Actin

Supplementary Figure 18. Original full blots for the cropped images shown in Supplementary Fig.11a

a) PI3Kδ and β-actin
