# Supplementary material for: PI3Kδ activity controls plasticity and discriminates between EMT and stemness based on distinct TGFβ signaling
Source: Commun Biol. 2022 Jul 25;5:740. doi: 10.1038/s42003-022-03637-w (PMC9314410; doi:10.1038/s42003-022-03637-w)
Supplement: Supplementary file 2 — Description of Additional Supplementary Files [file 42003_2022_3637_MOESM2_ESM.pdf]

Description of Additional Supplementary Files

File name: Supplementary Data 1
Description: Regulated genes in transcriptome from PI3Kδ overexpression in Huh7 cells versus control cells.

File name: Supplementary Data 2
Description: Regulated genes in transcriptome of mouse liver infected with AAV8-PIK3CD vector versus empty vector.

File name: Supplementary Data 3
Description: List of antibodies.

File name: Supplementary Data 4
Description: List of primers used for RT-qPCR

File name: Supplementary Data 5
Description: All source data for the graphs and charts presented in the main and supplementary figures.
