# Supplementary material for: PI3Kδ activity controls plasticity and discriminates between EMT and stemness based on distinct TGFβ signaling
Source: Commun Biol. 2022 Jul 25;5:740. doi: 10.1038/s42003-022-03637-w (PMC9314410; doi:10.1038/s42003-022-03637-w)
Supplement: Supplementary file 8 — Reporting Summary [file 42003_2022_3637_MOESM8_ESM.pdf]

nature research

Corresponding author(s): Ama Gassama-Diagne

Last updated by author(s): Jun 14, 2022

Reporting Summary

Nature Research wishes to improve the reproducibility of the work that we publish. This form provides structure for consistency and transparency in reporting. For further information on Nature Research policies, see our Editorial Policies and the Editorial Policy Checklist.

Statistics

For all statistical analyses, confirm that the following items are present in the figure legend, table legend, main text, or Methods section.

n/a	Confirmed	
☐	☒	The exact sample size (n) for each experimental group/condition, given as a discrete number and unit of measurement
☐	☒	A statement on whether measurements were taken from distinct samples or whether the same sample was measured repeatedly
☐	☒	The statistical test(s) used AND whether they are one- or two-sided<br>*Only common tests should be described solely by name; describe more complex techniques in the Methods section.*
☐	☒	A description of all covariates tested
☐	☒	A description of any assumptions or corrections, such as tests of normality and adjustment for multiple comparisons
☐	☒	A full description of the statistical parameters including central tendency (e.g. means) or other basic estimates (e.g. regression coefficient) AND variation (e.g. standard deviation) or associated estimates of uncertainty (e.g. confidence intervals)
☐	☒	For null hypothesis testing, the test statistic (e.g. F, t, r) with confidence intervals, effect sizes, degrees of freedom and P value noted<br>*Give P values as exact values whenever suitable.*
☐	☒	For Bayesian analysis, information on the choice of priors and Markov chain Monte Carlo settings
☐	☒	For hierarchical and complex designs, identification of the appropriate level for tests and full reporting of outcomes
☐	☒	Estimates of effect sizes (e.g. Cohen's d, Pearson's r), indicating how they were calculated

Our web collection on statistics for biologists contains articles on many of the points above.

Software and code

Policy information about availability of computer code

Data collection	BD Acuri C6, Leica LASX, Case viewer, Motic image plus 2.0
Data analysis	Microsoft Excel 2013, Graphpad Prism 8, R, Fiji-ImageJ, QuPath, FlowJo 10

For manuscripts utilizing custom algorithms or software that are central to the research but not yet described in published literature, software must be made available to editors and reviewers. We strongly encourage code deposition in a community repository (e.g. GitHub). See the Nature Research guidelines for submitting code & software for further information.

Data

Policy information about availability of data

All manuscripts must include a data availability statement. This statement should provide the following information, where applicable:
- Accession codes, unique identifiers, or web links for publicly available datasets
- A list of figures that have associated raw data
- A description of any restrictions on data availability

Raw data from affymetrix microarray have been deposited under the following code: GSE128022 (Huh7 transcriptomes)

Field-specific reporting

Please select the one below that is the best fit for your research. If you are not sure, read the appropriate sections before making your selection.

☒ Life sciences ☐ Behavioural & social sciences ☐ Ecological, evolutionary & environmental sciences

For a reference copy of the document with all sections, see nature.com/documents/nr-reporting-summary-flat.pdf

Life sciences study design

All studies must disclose on these points even when the disclosure is negative.

Sample size	Sample size for each experiment is indicated in the figure legends; No statistical methods were used to predetermine sample size
Data exclusions	No data were excluded from the analysis
Replication	All the statistical analysis were performed on at least 2 biologically independent replicates.
Randomization	N.A.
Blinding	N.A.

Reporting for specific materials, systems and methods

We require information from authors about some types of materials, experimental systems and methods used in many studies. Here, indicate whether each material, system or method listed is relevant to your study. If you are not sure if a list item applies to your research, read the appropriate section before selecting a response.

Materials & experimental systems

n/a	Involved in the study	
☐	☒	Antibodies
☐	☒	Eukaryotic cell lines
☒	☐	Palaeontology and archaeology
☐	☒	Animals and other organisms
☒	☐	Human research participants
☒	☐	Clinical data
☒	☐	Dual use research of concern

Methods

n/a	Involved in the study	
☒	☐	ChIP-seq
☐	☒	Flow cytometry
☒	☐	MRI-based neuroimaging

Antibodies

Antibodies used	Zona-occludens 1 (Invitrogen, 33-9100, mouse anti-human), Cytokeratin 19 (DAKO, M0888, mouse anti-human), Ep-CAM (Sigma, SAB4200473, mouse anti-human), PI3Kδ (abcam, ab32401, Rabbit anti-human), PI3Kδ (Santa Cruz, Sc 7176, Rabbit anti-human), laminin (Sigma, L9393, Rabbit anti-human), Notch2 (abcam, ab8926, Rabbit anti-human), Notch3 (abcam, ab23426, Rabbit anti-human), alexa fluor coupled Secondary antibody(life technologies, Donkey), E-cadherin (BD Biosciences, 610180, Mouse anti-human), Vimentin (Millipore, CBL202, Mouse anti-human), β-Actin (Cell signaling, 13E5, Rabbit anti-human), Src (abcam, ab47411, Rabbit anti-human), GP135 (George Ojakian, University of New York Downstate Medical Center,USA, Mouse anti-human), Albumin (Santa Cruz, sc-271605, Mouse anti-human) p-ERK 44/42 (Cell signaling, 9101S, Rabbit anti-human), P-Src (abcam, ab47411, Rabbit anti-human), Hoechst (life technologies, 34580), Phalloïdin (life technologies, A22283), p-akt (Cell signaling, 587f11, Mouse anti-human), p-smad2 (Cell signaling, D27F4, Rabbit anti-human)
Validation	All these antibodies were already validated and used in a published article on human cells lines

Eukaryotic cell lines

Policy information about cell lines

Cell line source(s)	Huh7, HepG2 and Hep3B from ATCC, MDCK (Madin-Darby Canine Kidney) from Keith Mostov laboratory, UCSF, San Francisco, hESC (human embryonic stem cells) from WiCell , HepaRG from Biopredic
Authentication	*Describe the authentication procedures for each cell line used OR declare that none of the cell lines used were authenticated.*
Mycoplasma contamination	All the cell lines were negative for mycoplasma contamination

Commonly misidentified lines (See ICLAC register)

Name any commonly misidentified cell lines used in the study and provide a rationale for their use.

Animals and other organisms

Policy information about studies involving animals; ARRIVE guidelines recommended for reporting animal research

Laboratory animals	C57BL/6 mice, male, 8 weeks
Wild animals	The study did not involved wild animals
Field-collected samples	C57BL/6 mice were maintained under specific pathogen-free conditions, and food and water were provided ad libitum. Mice were injected in the tail vein at 8 weeks after birth using pAAV TBG m PI3Kδ and pAAV TBG EGFP adenovirus (1011 particules / mouse) and sacrificed at 12 weeks after birth.
Ethics oversight	The University of Liege ethical committee approved all protocols under number 1738.

Note that full information on the approval of the study protocol must also be provided in the manuscript.

Flow Cytometry

Plots

Confirm that:

- [x] The axis labels state the marker and fluorochrome used (e.g. CD4-FITC).
- [x] The axis scales are clearly visible. Include numbers along axes only for bottom left plot of group (a 'group' is an analysis of identical markers).
- [x] All plots are contour plots with outliers or pseudocolor plots.
- [x] A numerical value for number of cells or percentage (with statistics) is provided.

Methodology

Sample preparation	Cells were dissociated using Accutase followed by a neutralization step with culture media. 1/100 of primary antibody was added for 1 hour, and after washing the cells were stained and incubated with the secondary antibody for 1 hour. Fluorescence intensity was measured by flow cytometry with BD Accuri C6 plus software. Data Analysis was performed using the FlowJo software.
Instrument	BD Acuri C6,
Software	FlowJo 10
Cell population abundance	*Describe the abundance of the relevant cell populations within post-sort fractions, providing details on the purity of the samples and how it was determined.*
Gating strategy	*Describe the gating strategy used for all relevant experiments, specifying the preliminary FSC/SSC gates of the starting cell population, indicating where boundaries between "positive" and "negative" staining cell populations are defined.*

- [x] Tick this box to confirm that a figure exemplifying the gating strategy is provided in the Supplementary Information.
